# Pseudoautosomal Region 1 Overdosage Affects the Global Transcriptome in iPSCs From Patients With Klinefelter Syndrome and High-Grade X Chromosome Aneuploidies

**DOI:** 10.3389/fcell.2021.801597

**Published:** 2022-02-03

**Authors:** Veronica Astro, Maryam Alowaysi, Elisabetta Fiacco, Alfonso Saera-Vila, Kelly J. Cardona-Londoño, Riccardo Aiese Cigliano, Antonio Adamo

**Affiliations:** ^1^ Biological and Environmental Science and Engineering Division, King Abdullah University of Science and Technology, Thuwal, Saudi Arabia; ^2^ Sequentia Biotech SL, Barcelona, Spain

**Keywords:** Klinefelter syndrome, high-grade X aneuploidies, X chromosome inactivation, escape genes, pseudoautosomal region, induced pluripotent stem cells

## Abstract

Klinefelter syndrome (KS) is the most prevalent aneuploidy in males and is characterized by a 47,XXY karyotype. Less frequently, higher grade sex chromosome aneuploidies (HGAs) can also occur. Here, using a paradigmatic cohort of KS and HGA induced pluripotent stem cells (iPSCs) carrying 49,XXXXY, 48,XXXY, and 47,XXY karyotypes, we identified the genes within the pseudoautosomal region 1 (PAR1) as the most susceptible to dosage-dependent transcriptional dysregulation and therefore potentially responsible for the progressively worsening phenotype in higher grade X aneuploidies. By contrast, the biallelically expressed non-PAR escape genes displayed high interclonal and interpatient variability in iPSCs and differentiated derivatives, suggesting that these genes could be associated with variable KS traits. By interrogating KS and HGA iPSCs at the single-cell resolution we showed that PAR1 and non-PAR escape genes are not only resilient to the X-inactive specific transcript (XIST)-mediated inactivation but also that their transcriptional regulation is disjointed from the absolute XIST expression level. Finally, we explored the transcriptional effects of X chromosome overdosage on autosomes and identified the nuclear respiratory factor 1 (NRF1) as a key regulator of the zinc finger protein X-linked (ZFX). Our study provides the first evidence of an X-dosage-sensitive autosomal transcription factor regulating an X-linked gene in low- and high-grade X aneuploidies.

## Introduction

Klinefelter syndrome (KS) is the most common sex chromosome aneuploidy in humans with a prevalence of 1:500–1:1,000 in males (Bojesen et al., 2003; Davis et al., 2016). KS is characterized by a 47,XXY karyotype in 80–90% of patients, and the remaining 10–20% of patients exhibit higher grade aneuploidies (e.g., karyotypes 48,XXXY, 48,XXYY, and 49,XXXXY), nowadays referred as high grade sex chromosome aneuploidies (HGAs), structurally abnormal X chromosomes (e.g., 47,iXq,Y), and mosaicism (karyotype 46,XY/47,XXY) (Forti et al., 2010). The pathophysiology of KS is highly variable among individuals. Common clinical manifestations include infertility, gynecomastia, tall stature, small testes, osteoporosis, and sparse body hair, whereas other features, such as cardiac structural abnormalities, metabolic disorders, type two diabetes, cancers, and intellectual disability, are less frequent (Swerdlow et al., 2001; Lanfranco et al., 2004; Bojesen et al., 2006; Groth et al., 2013; Bonomi et al., 2016). Notably, less prominent clinical symptoms are associated with milder genetic abnormalities, as in the case of mosaic patients (Samplaski et al., 2014). By contrast, severe neurodevelopmental and physiological disorders are associated with patients with HGAs (e.g., 49,XXXXY and 48,XXXY) (Tartaglia et al., 2011). The KS and HGAs etiopathology is attributable to sex chromosome non-disjunction during gametogenesis at meiosis I or II. The non-disjunction event results in gametes with abnormal sex chromosome numbers, which in turn leads to an XXY zygote. Nondisjunction during early mitotic division of the zygote results in a mosaic form of KS (Lanfranco et al., 2004; Bonomi et al., 2016).

The X-chromosome inactivation (XCI) mechanism physiologically compensates for the difference in dosage of X-linked gene copies between males and females (Augui et al., 2011; Disteche 2016). XCI is triggered by the long noncoding RNA (lncRNA) X-inactive specific transcript (*XIST*), which mediates the coating and silencing of the inactive X chromosome (Xi) in *cis* (Heard 2006; Payer and Lee 2014). The transcriptionally silenced X is accompanied by heterochromatin modifications and is cytologically detected as a condensed Barr body (Heard 2005; Hall and Lawrence 2011). This mechanism is also active in KS and HGAs patients (Froland and Skakkebaek 1971; Shamsuddin and Tang 1980; Brown et al., 1991; Kleinheinz and Schulze 1994; Schulz and Heard 2013). Importantly, a subset of X-linked genes, called “escape”, eludes the XCI mechanism and is actively transcribed from Xi (Tukiainen et al., 2017).

Pseudoautosomal regions 1 and 2 (PAR1 and PAR2) are two unique regions of the human genome located at the terminal ends of Xp and Xq spanning approximately 2.6 Mb and 320 kb, respectively (Freije et al., 1992; Rappold 1993; Helena Mangs and Morris 2007). PARs enable the pairing of sex chromosomes during meiosis. PAR1 is evolutionarily older than PAR2 and is conserved in most Eutherian mammals (Otto et al., 2011). The genes comprised within PAR1 are physiologically expressed from both X and Y chromosomes in males and escape X inactivation in females. PAR2 evolved after the divergence of humans and chimpanzees (Ciccodicola et al., 2000; Charchar et al., 2003; Hughes et al., 2010) and has only been identified in the human genome. PAR2 only contains four genes; *VAMP7* and *SPRY3*, which are more centromeric, are mostly inactive on the Y chromosome and subjected to inactivation on Xi, whereas *WASHP6* and *IL9R,* which are more telomeric, escape X-inactivation (D’Esposito et al., 1996; De Bonis et al., 2006). Although the biallelic expression of PAR1 genes has been conserved throughout evolution and correlates with a model of regionalized escape of X inactivation (Ciccodicola et al., 2000), the dual mechanism of regulation of the more recent PAR2 region is not yet fully understood. Therefore, the overdosage of PAR genes in KS and HGAs is expected to contribute substantially to the molecular landscape of the phenotype in patients with low and high-grade X chromosome aneuploidies.

Surprisingly, despite the high prevalence of KS, the few studies demonstrating a correlation between the overdosage of X-linked genes with the clinical features of KS are limited to 47,XXY patients and are restricted to specific tissue types (Viana et al., 2014; Zitzmann et al., 2015; Belling et al., 2017; Raznahan et al., 2018; Skakkebæk et al., 2018; Winge et al., 2018; Zhang et al., 2020). Moreover, a previous report described the derivation of human induced pluripotent stem cells (hiPSCs) from KS coupled with a thorough analysis of XCI status (Panula et al., 2019). However, the mild to high degree of X reactivation in these lines limits their use as *bona fide* cellular models of KS. We recently reported the first somatic cell reprogramming of high-grade X chromosome aneuploid fibroblasts and derived a unique cohort of iPSCs from patients with KS, including rare 49, XXXXY HGA and mosaic 47,XXY/46,XY individuals from whom we generated naturally isogenic 48,XXXY and 46,XY iPSCs (Alowaysi et al., 2020a; Alowaysi et al., 2020b; Alowaysi et al., 2020c; Fiacco et al., 2020; Fiacco et al., 2021). This cohort has been thoroughly characterized to assess pluripotency and genome integrity. Additionally, we assessed whether the supernumerary X chromosomes originated from aberrant chromosomal segregation during meiosis I or II, by combining karyotyping and short tandem repeat analysis (Alowaysi et al., 2020a; Alowaysi et al., 2020b; Alowaysi et al., 2020c; Fiacco et al., 2020; Fiacco et al., 2021).

In this study, we evaluated the gene dysregulation resulting from X dosage imbalance in pluripotency and during differentiation and demonstrated that the expression of genes within PAR1, but not PAR2, stoichiometrically mirrors X chromosome dosage across 47,XXY, 48,XXXY, and 49,XXXXY. By contrast, only a few non-PAR escape genes are biallelically expressed in the context of fully retained X inactivation. Moreover, using a single-cell RNA-sequencing approach, we showed that the expression of PAR1 and non-PAR escape genes in KS- and HGA-iPSCs is independent of *XIST* levels. Finally, we determined the impact of PAR1 overdosage on the global transcriptome and identified nuclear respiratory factor 1 (NRF1) as an X-dosage-dependent autosomal transcription factor (TF) that directly regulates the zinc finger protein X-linked (*ZFX*) gene in KS- and HGA-iPSCs.

## Materials and Methods

### Fibroblast Culture

The primary fibroblast cell lines, HM (GM00321), HB (GM00323), KS1 (GM00326), KS4 (GM03102), KS7 (GM00157), were obtained from Coriell Institute Repositories, New Jersey, United States. KS2 (FFF0312002) primary fibroblasts were obtained from Telethon Network of Genetic Biobank, Istituto Giannina Gaslini Rome, Italy. KS3 (CR0189M-6674), KS5 (GA00156M0-2276) and KS6 (P-C95121M-807) primary fibroblasts were obtained from Galliera Genetic Biobank, Genova, ITALY. All fibroblasts were cultured and expanded for at least three passages in high glucose DMEM (Thermo Fisher Scientific, Waltham, Massachusetts United States, Cat#61965-026) supplemented with 15% fetal bovine serum (FBS) (Thermo Fisher Scientific, Cat#16140071), 1% non-essential amino acids (Thermo Fisher Scientific, Cat#11140-035) and 1% penicillin-streptomycin (Thermo Fisher Scientific, Cat#15070063) before performing cell reprogramming.

### Human iPSC Culture and siRNA Transfection

The established hiPSC lines were cultured in feeder-free conditions on Vitronectin (VTN-N) (Thermo Fisher Scientific, Cat#A14700) coated plates in E8 medium (Thermo Fisher Scientific, Cat#A1517001) and passaged with Versene (Thermo Fisher Scientific, Cat#15040066) in E8 medium supplemented with RevitaCell™ (Thermo Fisher Scientific, Cat#A2644501) in 5% CO2 and 5% O2 at 37°C. For NRF1 silencing experiment iPSCs have been transfected with three independent siRNAs (50 nM; Supplementary Table S13) using RNAiMAX Lipofectamine (Thermo Scientific). Cells were incubated in E8 medium for 2 days after transfection before collection for RNA extraction.

### H3K27Me3 Immunostaining

iPSCs plated on vitronectin-coated coverslips were used for immunofluorescence 48–72 h after seeding. Briefly, cells were fixed for 12 min with 3% paraformaldehyde at room temperature, permeabilized with 0.25% Triton-X100 in PBS, incubated overnight with the H3K27Me3 primary antibody (Supplementary Table S14), washed, incubated with the Alexa Fluor488 goat-anti-mouse secondary antibody, and mounted with ProLong Gold antifade mounting solution (Thermo Scientific) for confocal microscopy acquisition.

### Definitive Endoderm Differentiation

Differentiation of hiPSCs toward definitive endoderm was performed according to PSC Definitive Endoderm Kit (Thermo Fisher Scientific, Cat#A3062601) following the manufacturer instructions. Briefly, hiPSCs were detached using Versene and seeded on VTN-N-coated plates in presence of E8 medium and RevitaCell™. After overnight culture, Medium A was added to 15–30% confluent cells for 24 h to induce endoderm differentiation, then cells were treated with Medium B for 24 additional hours and collected for further analysis.

### RNA Isolation, cDNA Preparation, and Reverse-Transcription (RT)-PCR

For gene expression analyses, total RNA was extracted using the RNeasy Mini Kit (QUIAGEN, Venlo, Netherlands, Cat#74106) according to manufacturer’s instructions. DNase treatment was performed using RNase-free DNase Set (QUIAGEN, Cat#79254). cDNA was synthetized with the SuperScript VILO IV cDNA Synthesis Kit (Thermo Fisher Scientific, Cat#11754050). Gene expression was determined by real time qPCR on a QuantStudio 3 Real-Time PCR System (Thermo Fisher Scientific) using TaqMan™ Fast Advanced Master Mix (Thermo Fisher Scientific, Cat#4444964) and 10 μM TaqMan® Gene Expression Probes (Supplementary Table S13). Individual gene expression was normalized on Tata Binding Protein (TBP).

### RNA Fluorescence *in situ* Hybridization

The RNA-FISH protocol was modified from the Stellaris (Biosearch Technologies, Hoddesdon United Kingdom) protocol for adherent mammalian cells. hiPSCs were grown on VTN-N (Thermo Fisher Scientific, Cat#A14700) -coated coverslips and fixed with 3.7% PFA (Sigma-Aldrich, Cat#F8775) at room temperature (RT) for 10 min, washed in 1X PBS (Thermo Fisher Scientific, Cat#14190250) and permeabilized with 0.25% Triton X-100 (VWR, Cat#A16046-AE) for 10 min. Fixed cells were incubated with 10% formamide (Sigma-Aldrich, Cat#F9037) in 2X saline-sodium citrate (SSC) (Thermo Fisher Scientific, Cat#BP132520) for 5 min and then in 125 nM Stellaris probes diluted in hybridization buffer for 8 h at 37°C. Human XIST probes labeled with CAL Fluor Red 610 (Biosearch Technologies) were used. Forty-five oligonucleotides designed against unique intronic sequences of human KDM6A labeled with Quasar 670 were obtained from Stellaris (BioSearch Technologies) (Supplementary Table S14). After hybridization, cells were incubated in 10% formamide (Sigma-Aldrich, Cat#F9037) in 2X SSC (Thermo Fisher Scientific, Cat#BP132520) for 30 min and then nuclei stained with DAPI (Sigma-Aldrich, Cat##D9564) for 30 min at 37°C. Coverslips were then washed in 2X SSC (Thermo Fisher Scientific, Cat#BP132520) for 5 min and mounted in ProLong® Gold Antifade Mountant (Thermo Fisher Scientific, Cat#P36934).

### Microscopy and Image Analysis

Bright-field images were acquired using a phase-contrast EVOS^TM^ XL Core microscope (Thermo Fisher Scientific) equipped with 10X and 20X objectives (Olympus). Immunostainings for pluripotency markers, H3K27me3, and RNA-FISH images were acquired using an EVOS^TM^ FL Auto 2 Imaging System (Thermo Fisher Scientific) using a 1.30 NA/40X oil or 1.42 NA/60X oil immersion objectives (Olympus). RNA FISH and H3K27me3 images were acquired as Z-stacks of 0.3 µm steps, processed by maximum intensity projections, and analyzed with ImageJ software (Open Source, https://imagej.net). At least 600 single cells spread over 12 fields of views were acquired for each cell line. The number of nuclei/field was defined by DAPI-positive cells, and the percentage of positive signals for XIST and KDM6A RNA-FISH, as well as H3K27me3, was calculated. Identical exposure settings were applied for all image acquisitions. RNA-FISH experiment was performed in triplicates and immunostaining of H3K27me3 in duplicates.

### Bulk RNA-Seq Library Preparation and Sequencing

RNA libraries were generated using the human mRNA TruSeq Stranded library preparation KIT from Illumina (Illumina, San Diego, California United States, Cat#20020594), and profiled using a HiSeq4000 system with 150 bp paired-end sequencing method. An average of 38M reads were obtained for each sample. Samples with less than 18M input reads and lower than 75% assigned reads were removed from the analysis (Supplementary Table S12).

### RNA-Seq Data Profiling

To analyze RNA-Seq data the Artificial Intelligence RNA-Seq (AIR) Software as a Service (SaaS) platform (https://transcriptomics.cloud) (Vara et al., 2019) was used. RNA-Seq data validation, pairing and FastQC quality control was followed by trimming using BBDuk (Bushnell et al., 2017) by setting a minimum read length of 35 bp and a minimum Phred-quality score of 25. After trimming quality control, high quality reads were mapped against the reference genome (GRCh38/Ensembl release 95) using the STAR end-to-end alignment method (Dobin et al., 2012) and gene expression quantification performed with featureCounts (Liao et al., 2014). The automated statistical analysis by AIR included a cleaning step for lowly expressed genes using HTSFilter (Rau et al., 2013). Genes with expression levels below the cut off (FPKM < 0.5) were excluded from the analysis in agreement with EMBL-EBI guidelines (https://www.ebi.ac.uk/gxa/.html). For the identification of differentially expressed genes (DEGs) the statistical methods edgeR (Robinson et al., 2009) was used. Data normalization was performed with the Trimmed Mean of M-values (TMM) method. Genes were considered differentially expressed if they had FDR < 0.05, a Log_2_FC > 0.25 for upregulated and a Log_2_FC < −0.25 for downregulated genes. Two to four RNA-Seq replicates per clone were analyzed. Plots generated for data interpretation were the Principal Component analysis (PCA) for sample clustering, heatmaps showing the color-coded FPKM expression of selected groups of genes (Metsalu and Vilo 2015) and Box Plot to compare single gene expression levels. EnrichR (Chen et al., 2013) was used to conduct GO enrichment analysis to identify the biological processes and DisGeNET v7.0 (Piñero et al., 2015; Piñero et al., 2016; Piñero et al., 2019) as database for genes associated to human diseases on the commonly X-linked DEGs from the comparisons 47,XXY vs. 46,XY and 48,XXXY-49, XXXXY vs. 46, XY at DE stage. Significance for biological processes and gene-disease associations was set with Bonferroni test for corrected *p* values < 0.05.

### Single Cell RNA-Seq Library Preparation

Viable cells were counted with Trypan Blue (Thermo Fischer Scientific, Cat#T8154) solution, and for each cell line, 1,000–2,000 viable cells were loaded into a chip and processed with the Chromium Single Cell Controller (10X Genomics, Pleasanton, California United States, estimated recovery: 1,500 cells/sample). Single-cell RNA-Seq libraries were prepared using the Single Cell 3′ GEM, Library and Gel Bead Kit V3 (10× Genomics, Cat#PN-1000075) according to manufacturer instructions. Briefly, single cells were partitioned into individual Gel Beads-in-emulsion (GEMs) and the RNA obtained from lysed cells was barcoded through reverse transcription. Each cell is encapsulated in a gel bead that contains a unique 14- base pair (bp) molecular barcode, a 10-bp randomer to index molecules (unique molecular identifier, UMI), and an anchored 30-bp oligo-dT to prime polyadenylated RNA transcripts. DynaBeads® MyOne Silane Beads (Thermo Fischer Scientific, Cat#37002D) were used to purify the resulting barcoded cDNA that was subsequently amplified *via* PCR (12-14 cycles, according to the available cDNA quantity). Libraries were then checked and quantified *via* the Agilent 2100 Expert Software. The resulting libraries were sequenced across one lane using an Illumina Hiseq4000 machine (Supplementary Table S12).

### Single Cell RNA-Seq Data Profiling

The pre-processing of single cell RNA-Seq data that includes the identification of cell barcodes and the steps of UMI detection, extraction and processing was performed using UMI-tools 1.0.1 (Smith et al., 2017). Read quality control (QC) analyses and filtering of high-quality reads, were executed using FastQC and BBDuk (Bushnell et al., 2017) with default options of minimum size 35 bp and minimum quality score 25 to perform adapter and quality trimming. To align the filtered reads to a human genome reference (GrCh38 release 91) STAR 2.7.3a (Dobin et al., 2012) was used, while FeatureCounts 1.6.3 package (Liao et al., 2014) was used to assign reads to genes.

Data from single cell RNA-sequencing were then analyzed using the R packages: SC3 (Kiselev et al., 2017) for clustering methods, differential expression analysis and gene marker detection algorithm and Scater 1.16.0 (McCarthy et al., 2017) was used for expression data pre-processing, quality control, normalization and visualization through the t-distributed stochastic neighbor embedding (t-SNE) analysis. The package scImpute 0.0.9 (Li and Li 2018) was used to correct dropout values. For cell cycle analysis, cells were classified into cell cycle phases (G1, G2/M or S) based on the RNA-Seq gene expression data with the prediction method described by Scialdone et al. (2015) using the cyclone function and the pre-trained set of human gene marker pairs from the scran package v1.14.6 following published guidelines (Lun et al., 2016).

### Whole Exome Sequencing

Genomic DNA was extracted from hiPSC lines as described above. WES analysis was performed by Beijing Novogene Bioinformatics Technology, Co., Ltd. (Beijing, China). Target enrichment was performed to construct the exome library using the Agilent SureSelect Human All Exon V6 kit (Agilent Technologies, Inc., Santa Clara, CA, United States), according to manufacturer’s protocol, and sequenced on Illumina Platform. An effective coverage of around 100× was obtained for all samples

### Allele-specific Expression

To remove low-quality reads, FastQC and BBDuk were used with default options to perform adapter and quality trimming (minimum read size 35 bp and minimum quality score 20). High-quality reads were mapped to the human reference genome (hg19/GRCh37 version) using the Burrows-Wheeler Aligner (BWA). PCR duplicates were filtered out using Picard to ensure accurate alignment and variant calling rates. Variant calling was performed with GATK v4.1.4.1.

To control for possible RNA editing events, only variants identified by WES were analyzed with ASERead Counter. Variant calling of WES data was performed with Mutect2, and the resulting vcf files were filtered using FilterMutectCalls using the following options: 1) optimal F score was used as posterior probability threshold; 2) relative weight of recall to precision (f-score-beta) equal 1.0; 3) maximum false discovery rate equals 0.05; 4) 0.1 was the initial artifact probability threshold used in the first iteration; 5) mitochondria mode was set to false; 6) Maximum events in a single assembly region equal 2; 6) maximum alt alleles per site equal 1; 7) Minimum median mapping quality of alt reads equal 30; 7) Minimum median base quality of alt reads equal 20; 8) The maximum difference between median alt and ref fragment lengths equal 10,000; 9) Minimum median distance of variants from the end of reads equal 1, 10) Maximum fraction of non-ref bases in the pileup that are N equal Infinity; 11) Maximum fraction of alt reads that initially aligned outside the mitochondria equal 0.85; 12) Natual logarithm (ln) of the initial prior probability that a site has a somatic SNV equal to −13.815510557964275; 13) ln of the initial prior probability that a site has a somatic indel equal to -16.11809565095832; 14) ln of the initial prior probability that a called site is not a technical artifact equal to −2.302585092994046; 15) *p*-value threshold for standard artifact filter equal to 0.001; 16) Minimum number of reference bases in an STR to suspect polymerase slippage equal to eight; 17) frequency of polymerase slippage, in contexts where it is presumed, equal to 0.1; 18) On the second filtering pass, variants with the same PGT and PID tags as a filtered variant within this distance were filtered (--distance-on-haplotype 100); 19) Indels equal or greater than five were treated specially by the mapping quality filter (--long-indel-length 5). Next, the resulting vcf files were further filtered using the following criteria: 1) filter = “PASS”, meaning that all FilterMutectCalls filters were met; and 2) total reads greater than 10. The variants that satisfied all these criteria were used as a reference for the ASE analysis.

For ASE analysis, the counts of reads covering each allele at selected SNPs were obtained using ASERead Counter of GATK v4.1.4.1, adjusting the parameters to ensure adequate coverage and quality. We applied a highly stringent base quality parameter (higher than 30) to maintain higher confidence in the sequence of the reads and a more relaxed mapping quality parameter (MQ = 10) to maximize the detection of reads with a SNP that differs from the reference genome sequence in the specific position. Next, to further increase the accuracy of our analysis, we applied a read depth greater than six. Further downstream processing, we used filters by excluding: 1) sites that were intronic, and 2) were not uniquely mapping to genes (exception of *XIST*). For each SNP, reference allele expression was calculated as reference allele counts divided by total counts. SNPs were annotated as biallelic if reference allele expression fell within the range of 0.1–0.9 and monoallelic if it was below 0.1 or over 0.9. Genes were considered biallelic if one or more SNP annotated to that gene were biallelic.

### Moving Average Around the X Chromosome

Healthy male (XY) samples were used as reference to calculate the median FPKM level of each gene (male median) after removing non-expressed genes as previously described (Bar et al., 2019). Next, for every sample, the FPKM level of each gene was divided by the male median. These fold changes were then plotted in a moving average plot using a loess fit adjusting the span to 0.45, which determines the sliding window size as a proportion of observations in the data set.

MM (Male median) = Median (FPKM in reference XY samples) of a specific gene

FC (Fold Change) = FPKM in each sample/MM of a specific gene

### FPKM of X Expressed Genes and X:A Ratio

The mean FPKM of X expressed genes was calculated by averaging the expression of all X chromosome genes (FPKM > 0.5). Similarly, the X:Autosome (X:A) FPKM ratio was calculated dividing the mean FPKM of X chromosome by the average expression of autosomal genes for each sample. Both values are represented as karyotype means:

MF (mean FPKM) = Sum (FPKM expression)/Number of genes in chromosome

X: A FPKM ratio = X chromosome-MF/Average (Autosome-MF)

### WGCNA Analysis

To identify modules of co-expressed genes, we performed weighted-gene co-expression network analysis (WGCNA package, version 1.49) (Langfelder and Horvath 2008) using only genes with detectable expression (at least one count per million reads in at least four samples, 15,669 genes). The filtered matrix of counts was normalized by “variance Stabilizing Transformation” (DESeq2 package, v1.26.0) (Love et al., 2014). A signed network (soft thresholding power set to 20) was built using the normalized counts to detect modules of co-expressed genes. Modules with similar expression profiles were further grouped into super modules. Finally, Gene Ontology Enrichment Analysis (GOEA) was performed with hypergeometric test (phyper function from the stats R package). The *in silico* transcription factor binding site (TFBS) prediction analysis was performed with CiiiDER (Gearing et al., 2019) using the human GRCh38/Ensembl 95 as the reference genome. Analyses were performed on the promoter sequences using deficits of 0.15 with the 2020 JASPAR core non-redundant vertebrate matrices (Fornes et al., 2020). All promoter regions were defined as spanning –1,500 bases to +500 bases relative to the transcription start site. The query gene list was built using genes from the ten most significant supermodules of the WGCNA analysis. The background gene list was derived from lowly expressed transcripts (Log2 Fold change < |0.05|). Fisher’s exact test was used and gene coverage *p*-value <0.05 was considered significant.

### Statistical Analysis

Where indicated, two-way ANOVA and Fisher’s exact statistical analysis were used to test the significance between more than two groups. Alternatively, the nonparametric Student’s *t*-test, Mann-Whitney U, and Kruskal-Wallis statistical tests were used. For comparative analyses we applied the Pearson’s correlation coefficient. Median or mean values ± std are shown. A Bonferroni correction was applied to the *p-value* from multiple comparisons. *p < 0.05; **p < 0.01; ***p < 0.001.

## Results

### X Chromosome Inactivation Is Retained in KS- and HGA-iPSCs

**FIGURE 1:**
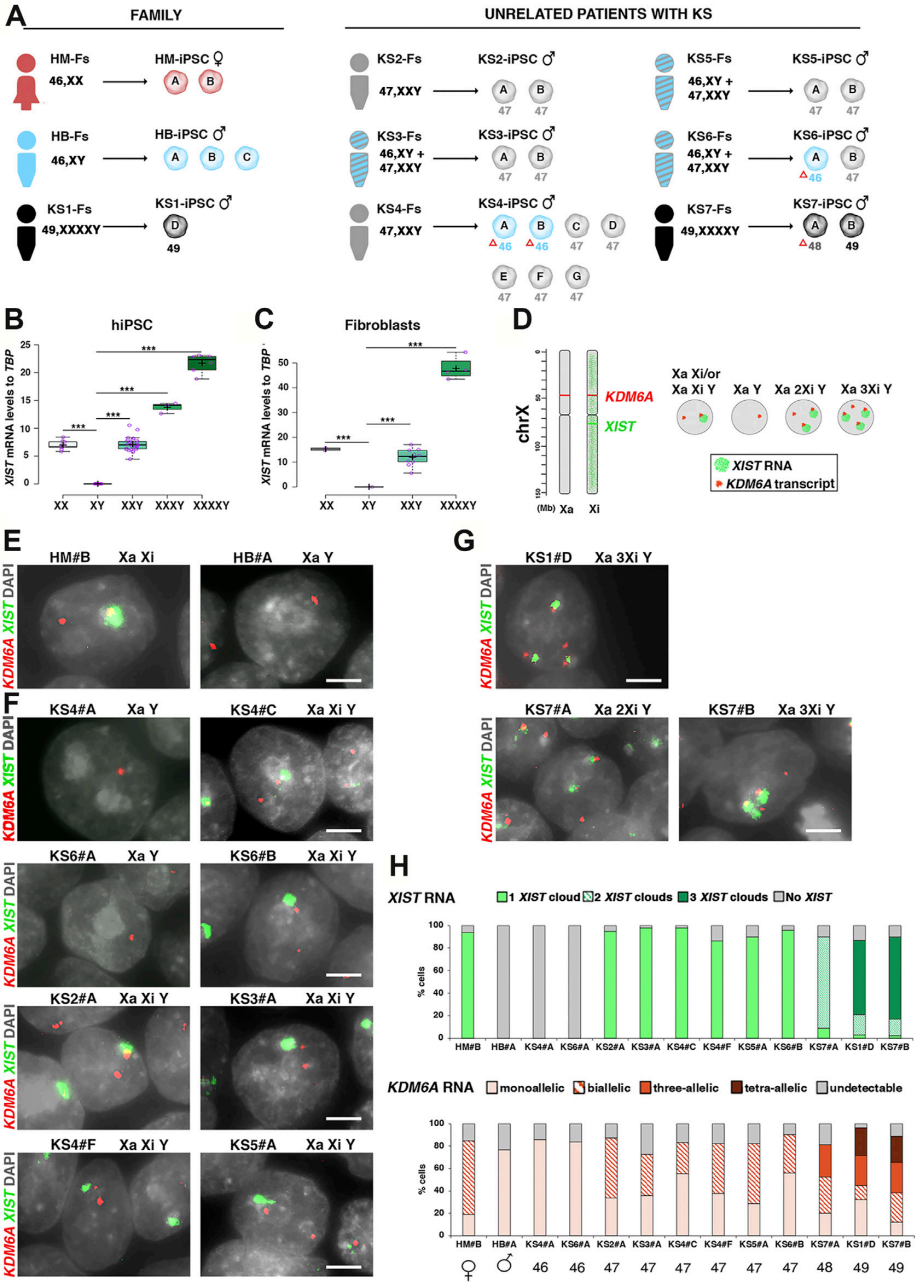
Patient cohort description and XIST dosage validation. **(A)** Cohort of recruited patients with KS and HGA, including fibroblasts and independent hiPSC clones derived from each patient and healthy controls. Each karyotype is specified by color: red, healthy mother; blue, healthy brother and isogenic 46,XY; black, 49,XXXXY and 48,XXXY; gray, non-mosaic 47,XXY; blue with gray stripes, mosaic 47/46,KS. The red triangle (Δ) indicates spontaneous X chromosome loss during reprogramming or naturally isogenic lines. **(B)** Box plot showing XIST expression in hiPSCs **(C)** and fibroblasts for the indicated karyotypes measured by Taqman quantitative polymerase chain reaction (q-PCR). Each purple dot represents a q-PCR expression analysis from at least three (hiPSCs) and two (fibroblasts) independent experiments. Each biological replicate is defined as an independent RNA sample obtained at consecutive passages from the same iPSC clone or from independent iPSC clones with the same karyotype. Centerlines show the medians; box limits indicate the 25th and 75th percentile. Whiskers extend 1.5 times the interquartile range from the 25th and 75th percentiles. In **(B)** *n* = 9, 9, 36, 3, and 6 sample points; in **(C)** *n* = 2, 2, 10, and 4 sample points. Student’s t-test, *p < 0.05; **p < 0.01, and ***p < 0.001. **(D–H)** Characterization of X chromosome inactivation (XCI) in KS- and HGA-iPSCs by RNA-FISH. **(D)** Schematic showing the localization of *XIST* and *KDM6A* genes on the X chromosome and *XIST* RNA coating (left). Model for XCI in euploid and aneuploid KS and HGA cells (right). **(E–G)** Representative RNA-FISH images of XIST-mediated X chromosome silencing (green) and KDM6A (red) mono-, bi-, tri-, and tetra-allelic expression in healthy iPSCs and in KS- and HGA-iPSCs. DNA was stained with DAPI (gray). Scale bar = 50 µm. **(H)** Percentage of *XIST* clouds and *KDM6A* signals counted in approximately 600 nuclei for the indicated iPSC lines.

The prerequisite for using KS- and HGA-iPSCs as a powerful *in vitro* disease model is the maintenance of the extra X chromosome/s in an inactive state. However, a progressive *XIST* loss has been reported in female human embryonic stem cells (hESCs) (Silva et al., 2008), possibly because of high passage numbers or culture conditions. This phenomenon is considered the first sign of a process called “X erosion”, which progressively leads to X reactivation (Mekhoubad et al., 2012; Nazor et al., 2012; Vallot et al., 2015; Patel et al., 2017). The mechanism of X erosion in female pluripotent stem cells (PSCs) has been debated in recent years, and a consensus has not yet been reached (Anguera et al., 2012; Mekhoubad et al., 2012; Vallot et al., 2015; Patel et al., 2017; Bar et al., 2019). Therefore, we analyzed the iPSC cohort (Figure 1A; Supplementary Table S1) at intermediate passages (p10–p14) and in fibroblasts from patients to determine *XIST* expression. Our results indicated that *XIST* expression stoichiometrically mirrors the X dosage in hiPSCs and fibroblasts (Figures 1B,C). Similarly, the expression of *JPX*, a master regulator of *XIST*, also reflects the X dosage (Supplementary Figures S1A,B). To further validate the X inactivation status at the single-cell level, we used an RNA-fluorescence *in situ* hybridization (FISH) approach to probe *XIST* and the nascent transcript of the non-PAR escape gene *KDM6A* as readouts for the inactivated and total number of X chromosomes per nuclei, respectively (Figure 1D). Our data showed that the number of *XIST* clouds in each iPSC line follows the “Ohno’s *n*-1 rule” (Ohno 1967), thus demonstrating that X inactivation is stably maintained, regardless of the number of extra Xs in hiPSCs (Figures 1E–H and Supplementary Table S2). This observation was supported by immunostaining for histone H3 lysine 27 trimethylation (H3K27me3), a repressive mark deposited by the PRC complex on the inactivated X chromosome (Plath et al., 2003) (Supplementary Figures S1C–H; Supplementary Table S2).

**FIGURE 2:**
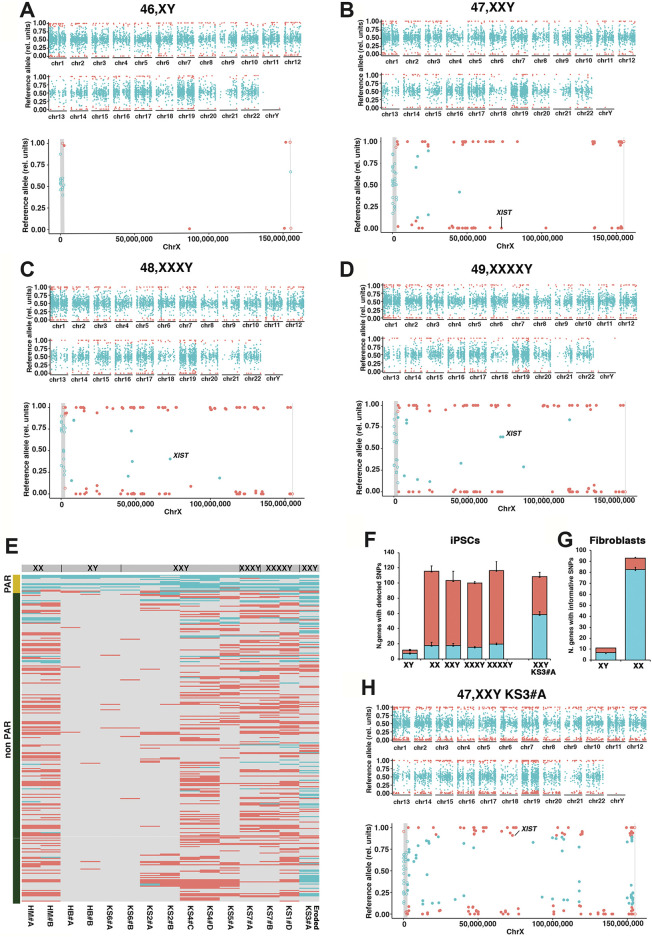
Allele-specific expression (ASE) profiles of cases and controls iPSCs highlighting the tight retention of X chromosome inactivation. **(A–D)** Scatter plot profiles of coupled WES analysis and allele-specific RNA-Seq analysis performed on autosomal (upper panel) and X chromosomes (lower panel) showing the mono- (orange dots) or biallelic (light blue dots) gene expression status in four representative karyotypes. Gray rectangles indicate PAR1 and PAR2 regions, respectively. Solid dots indicate non-PAR genes; open dots show PAR genes. *XIST* allele-specific expression status is displayed for each sample. **(E)** Heatmap of mono- (orange) and biallelically expressed (light blue) genes in the PAR and non-PAR regions for the indicated iPSCs. The nonparametric Mann-Whitney tests showed the statistically significant differences between the active/inactive status of PAR versus non-PAR genes: *p* = 1.57e-12 for all genotypes, *p* < 2.22e-16 for the KS3#A eroded iPSCs. **(F,G)** Graphs showing the number of biallelic and monoallelic genes with at least one informative SNP for each genotype in iPSCs **(F)** and fibroblasts **(G)**. Bars are medians ± standard deviations of two to three biological replicates for each sample. Two replicates of the 47,XXY KS3#A mildly eroded sample are shown. **(H)** Scatter plot profiles of the eroded 47, XXY KS3#A iPSCs sample.

Finally, to query the status of X inactivation at high resolution, we performed whole-exome sequencing (WES) followed by allele-specific expression (ASE) analysis to assess the coverage of expressed single nucleotide polymorphisms (SNPs) over the X. We observed that biallelically expressed SNPs are spread equally over autosomal chromosomes. By contrast, the distribution of almost all biallelically expressed SNPs on X is restricted to the PAR1 region (Figures 2A–E). Furthermore, the number of biallelic genes on X does not increase proportionally to the aneuploidy grade, indicating that the efficiency of X inactivation is equivalent in all karyotypes regardless of the number of Xs (Figures 2A–E; Supplementary Table S3). In the context of tight X inactivation retention, we found that a subset of PAR genes and the two non-PAR escape *KDM6A* and *PUDP* are consistently detected as expressed by Xa and Xi (Supplementary Figure S2A). Furthermore, the number of genes escaping X inactivation in hiPSCs, excluding PAR, is relatively low (Figures 2E,F; Supplementary Figure S2A) compared with a previously published dataset obtained from differentiated tissues, hESCs, or hiPSCs with mild to severe X chromosome erosion (Vallot et al., 2015; Patel et al., 2016; Cantone et al., 2017; Tukiainen et al., 2017; Garieri et al., 2018; Panula et al., 2019). An exception to the retained X inactivation state in our iPSC cohort is represented by the 47, XXY KS3#A iPSC line, which shows a mild X erosion (Figures 2F,H) despite *XIST* clouds and H3K27me3 validation (Figures 1F,H; Supplementary Figures S1G,H). In this sample, we detected approximately two to three times more X-linked biallelically expressed genes (*n* = 57) than any other KS- or HGA-iPSC clone (Figures 2F,H). This value is similar to that obtained for female fibroblasts (Figure 2G). In female fibroblasts approximately 89% of the X-linked genes are expressed from both alleles due to random X inactivation. (Plenge et al., 2002; Shvetsova et al., 2019). Moreover, to ensure the clonality of the eroded line, we confirmed that *XIST* is monoallelically expressed, thus excluding the possibility of a mosaic X inactivation (Figure 2H). Importantly, we detected similar average expression levels, SNP coverage, and assigned number of reads when comparing biallelic and monoallelic genes on autosomal and sex chromosomes in all samples (Supplementary Figures S2B,C; Supplementary Table S12). These findings rule out the possibility that the limited number of biallelically expressed genes on X would be an artifact resulting from a low average expression level of non-PAR escape versus PAR and autosomal genes. Taken together, these results demonstrate that KS- and HGA-hiPSCs could be maintained in culture for several passages without altering the XCI status. Additionally, the observation that X-linked biallelic genes are mostly restricted to the PAR1 region indicates that the transcriptional contribution of PAR1 genes to KS pathophysiology is more prevalent than that of escape genes during the early stages of human development.

### Transcriptomic Analysis Reveals Dosage-Dependent Proportional and Inversely Proportional Expression of X-Linked Genes

**FIGURE 3:**
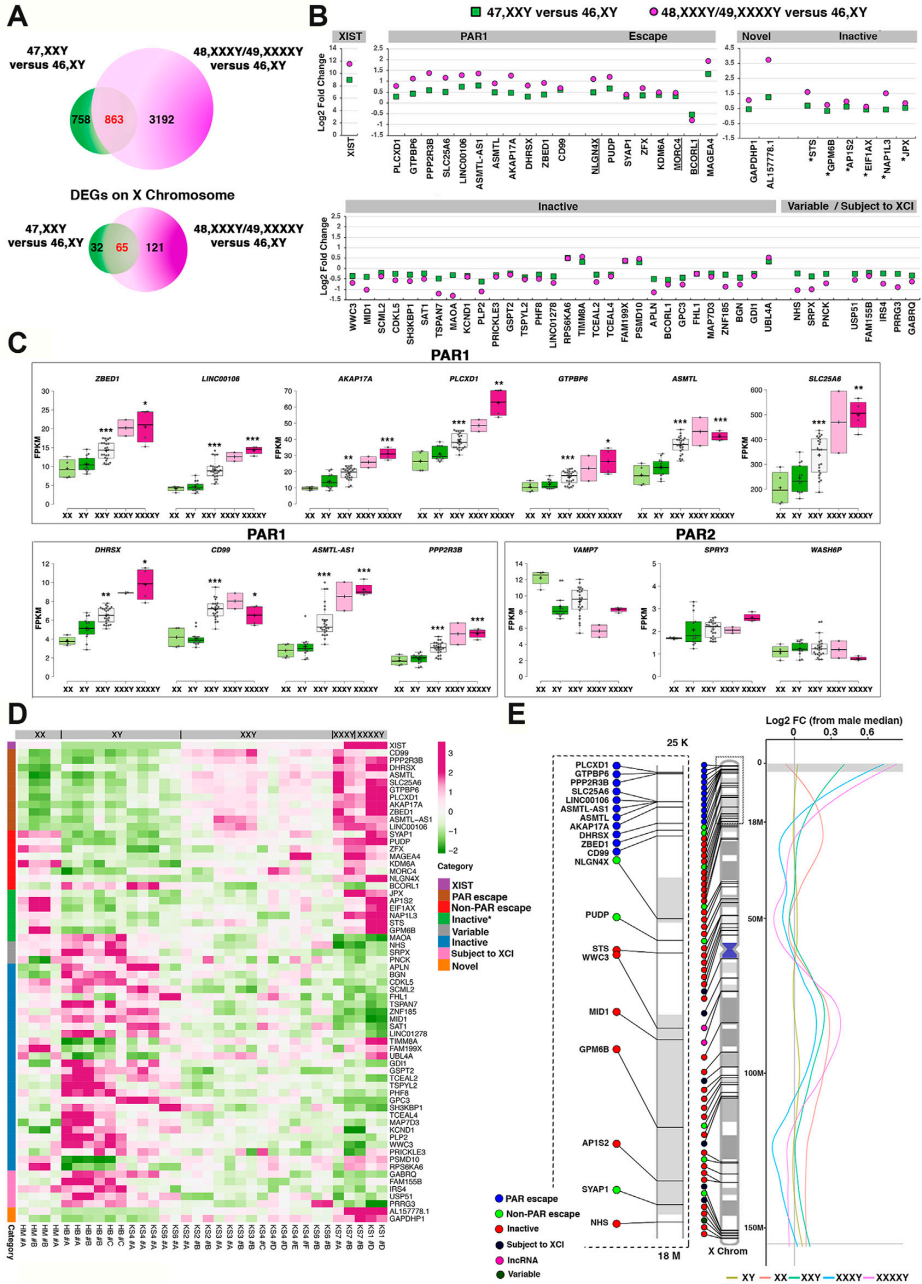
Transcriptomic profiling of KS- and HGA-iPSCs identifies proportional and inversely proportional X chromosome dosage-dependency of X-linked genes. **(A)** DEGs number and distribution across the comparisons 47, KS versus 46, XY and 48/49,XXXY/XXXXY versus 46, XY in the global transcriptome (upper Venn) or in the X chromosome (lower Venn). **(B)** Log_2_ fold change of shared dosage-sensitive X-linked DEGs. Six groups of DEGs are shown: genes from the PAR1 region, biallelic genes escaping Xi (Escape), monoallelic genes expressed from the active X (Inactive), and genes whose Xa/Xi status has never been described (Novel). Inactive DEGs labeled with asterisks have been previously described as escape genes. Variable and subject to XCI categories were assigned accordingly to previous studies. Underlined DEGs were previously described as inactive or variable (Tukiainen et al., 2017). **(C)** Boxplots showing the X dosage-dependent FPKM expression of genes from PAR1 and PAR2 regions. The significance between the comparison XXY (*n* = 24) versus XY (*n* = 13) and XXXXY (*n* = 4) versus XY (*n* = 13) was calculated using the two tailed Student’s t-test, *p < 0.05; **p < 0.01; ***p < 0.001. The significance for the comparison 48, XXXY (*n* = 2) versus 46, XY was not evaluated. **(D)** Heatmap showing the proportional and inversely proportional FPKM expression of shared X chromosome dosage-sensitive DEGs across the two comparisons, in PAR1, non-PAR escape, inactive, and novel categories in each RNA-Seq sample. Inactive DEGs marked with an asterisk have been previously described as Escape genes. DEGs were defined as variable and subject to XCI categories, according to previous studies (Tukiainen et al., 2017). **(E)** Left: Ideogram showing the position along the X chromosome of upregulated DEGs from the two comparisons in **(A)** included in the indicated categories, according to their Xa/Xi status. The dotted rectangle shows the magnified Xp chromosome area from 25 Kb to 18M. Right: Moving average line plot (loess fit, span = 0.45) along the X showing the log_2_ fold change (FC) from a control male. Gray horizontal boxes represent PAR1 and PAR2 limits. The gray vertical line represents no theoretical deviations from the control male.

By exploiting our iPSC lines carrying X chromosome aneuploidies representative of patients with 47,XXY, 48,XXXY and 49,XXXXY karyotypes, we tested whether transcriptional signatures associated with KS could be detected at the iPSC stage and whether the observed trends correlate with the reported increased phenotype severity in KS and HGA patients. To this end, we compared the identity and the average expression levels of X-linked genes in KS-iPSCs with those in healthy males or females. We found that the global transcriptional levels in our samples are comparable, regardless of sex or X aneuploidy grade (Supplementary Figure S3A and Mendeley data). Additionally, an X:autosomal ratio between 0.5 and 1 was observed for all karyotypes, indicating an equivalent dosage compensation to the autosomal content in our iPSC cohort (Lin et al., 2012; Pessia et al., 2012; Zhang et al., 2020) (Supplementary Figure S3B). We then performed two independent differential expression analyses comparing 47,XXY with 46 male transcriptomes (including isogenic 47 and 46 clones) and 48,XXXY/49,XXXXY with 46,XY male transcriptomes (Figure 3; Supplementary Figures S3C,D). Among 14,215 expressed genes in iPSCs, we identified 1,621 (47,XXY versus 46 males) and 4,055 (48,XXXY/49,XXXXY versus 46 males) differentially expressed genes (DEGs; Figure 3A and Mendeley Data). Notably, most DEGs identified in the low-grade KS comparison were also identified in the high-grade X aneuploidies. Focusing on the X-linked DEGs, we found 65 genes commonly dysregulated in both comparisons (Figure 3A and Supplementary Table S4). Importantly, the X-linked DEGs comprise 1) genes whose transcriptional dosage is attributable to both Xa and Xi (escaping X inactivation) and, therefore, directly related to the X copy number and, 2) genes that were expressed only from Xa and became up- or down-regulated as indirect targets. Among the 65 DEGs, 11 are located within the PAR1 region, eight are non-PAR escape genes, six are inactive genes reported as escape in previous studies (Balaton et al., 2015; Tukiainen et al., 2017), 36 have been previously reported to be inactive or variable (Tukiainen et al., 2017), five are subjected to XCI (Balaton et al., 2015), and two are X-linked genes for which the XCI status has not been previously described (named “novel”; Figure 3B; Supplementary Table S5). Importantly, we found that out of 25 known PAR1 genes located on the X chromosome, 15 are expressed in iPSCs. Approximately 75% of the expressed PAR1 genes display progressively increasing expression in XXY, XXXY, and XXXXY with three, four and five sex chromosomes respectively, compared with both female and healthy male controls (Figures 3C,D). Interestingly, we didn’t detect differentially expressed PAR2 genes in KS- and HGA-iPSCs compared with controls. Notably, the three PAR2 genes expressed in hiPSCs, *VAMP7*, *SPRY3* and *WASH6P*, do not mirror the X dosage (Figure 3C), similar to recent observations in primary human peripheral blood mononuclear cells (PBMCs) (Zhang et al., 2020). A possible explanation for the lack of dosage dependency for PAR2 could be related to the relatively low expression of PAR2 versus PAR1 genes in hiPSCs. Importantly, while all differentially expressed PAR1 are upregulated and mirror X dosage, non-PAR escape, variable, and inactive DEGs exhibit both proportional and inversely proportional expression trends (Figures 3B,D). These findings indicate that the downregulated DEGs could be indirect effects of the X dosage, consistent with two recent publications on X chromosome copy number variations (CNVs) (Raznahan et al., 2018; Zhang et al., 2020). Next, we analyzed the X chromosome density distribution of gene expression in KS- and HGA-iPSCs using a healthy male iPSC sample as a reference and we found that the terminal Xp arm, including but not limited to PAR1, is enriched with genes highly sensitive to X dosage (Figure 3E).

### The Expression of PAR and Non-PAR Escape Genes Is Regulated in an XIST-independent Manner

**FIGURE 4:**
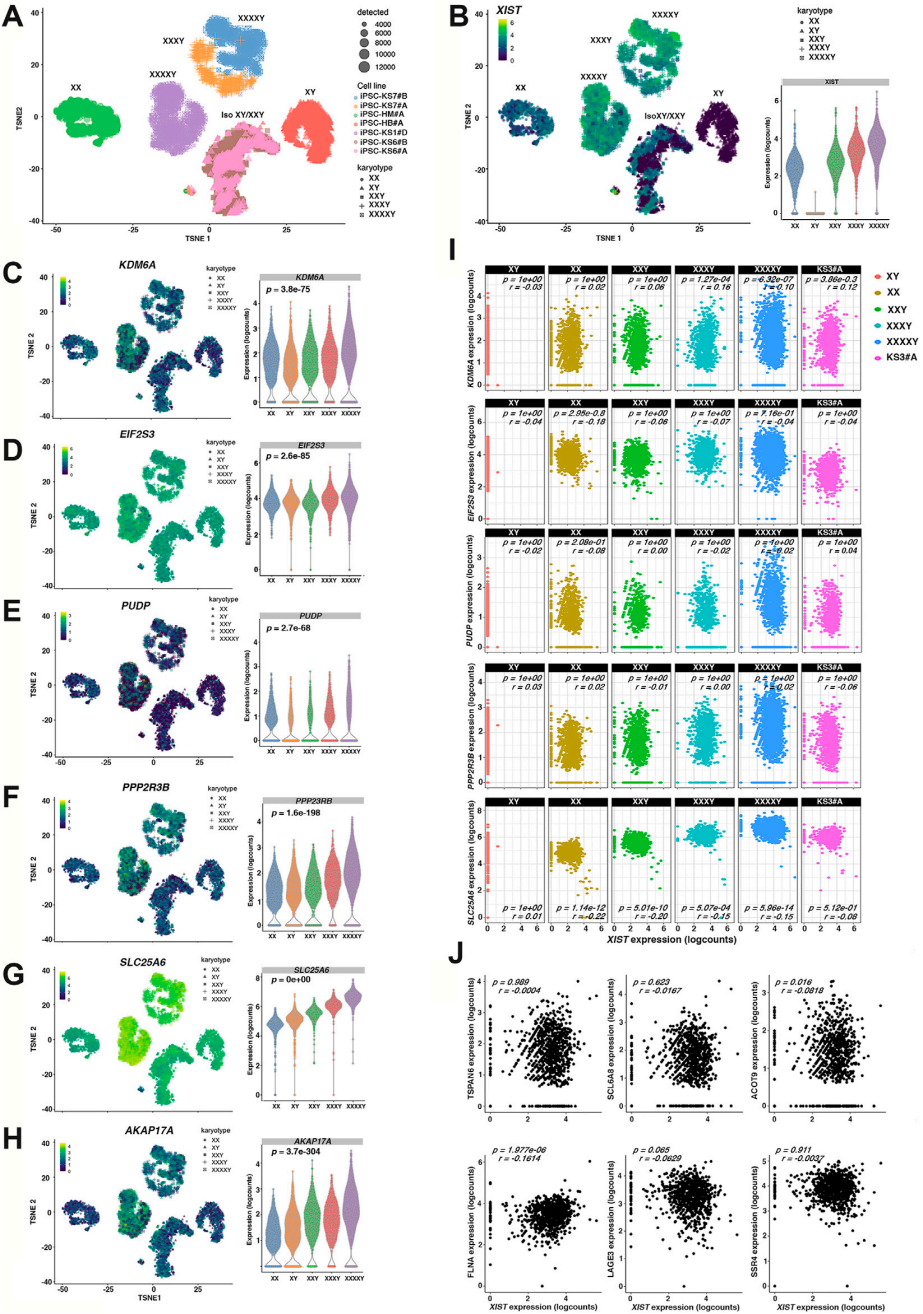
Single-cell RNA-sequencing of KS, HGA and controls iPSCs reveals homogenous XIST expression mirroring the X dosage and XIST-independency of escape genes on Xi. **(A)** t-Distributed stochastic neighbor embedding (t-SNE) plots of the whole-genome transcriptome for seven scRNA-Seq in 46,XX, 46,XY, 47,XXY, 48,XXXY, and 49,XXXXY hiPSCs. Circle sizes represent the number of detected genes/cells. A detailed description of the number of cells and the average number of genes detected for each sample is reported in Supplementary Table S12. Iso, Isogenenic cell line **(B)** Left: Expression profiles for the lncRNA *XIST*, right: Violin plot of XIST expression in the analyzed karyotypes. **(C–H)** Expression profiles for the indicated dosage-sensitive non-PAR escape **(C–E)** and PAR1 genes **(F–H)** were visualized by t-SNE (left) and Violin plots (right). The significance was calculated with the non-parametric Kruskal-Wallis statistical test. **(I)** Scatter plots showing the correlation between *XIST* expression levels (x-axis) and the expression value of the indicated PAR and non-PAR escape genes (y-axis) in each karyotype and the partially eroded 47,XXY (KS3#A) iPSC sample. **(J)** Correlation plot indicating the association of *XIST* expression with the levels of the predicted re-expressed genes in the partially eroded KS3#A sample. A parametric Pearson correlation test was applied. The significance was set at *p* > 0.05.

We then analyzed 47, 48, and 49, KS- and HGA-iPSCs at the single-cell level to directly correlate the expression of *XIST* with PAR and non-PAR escape genes. Recent single-cell studies on female fibroblasts (Garieri et al., 2018; Wainer Katsir and Linial 2019) have highlighted a certain degree of variability in the expression of genes that escape X inactivation, but a systematic correlation with *XIST* expression has not been thoroughly explored. We performed deep single-cell gene expression profiling in a cohort of seven non-eroded hiPSCs representing two 49 HGA, one 48 HGA, one 47 KS, two 46,XY, one 46, XX and one partially eroded 47,KS. Importantly, this cohort included the isogenic 48, HGA iPSC line derived from the 49, HGA patient KS7 and the isogenic healthy iPSC line derived from the mosaic patient KS6. The multidimensional reduction of global transcriptional profiles showed that each cell segregates by patient and karyotype origins (Figure 4A). The only exception to this observation is the mosaic patients, for whom we also analyzed the corresponding isogenic line. In this case, the samples cluster together with the isogenic counterpart indicating a prevailing genomic background effect over the X chromosome number (Figure 4A and Supplementary Figures S4A). However, when considering only the X chromosome transcriptome, the effect of X dosage becomes predominant (Supplementary Figures S4B). Consistent with our bulk RNA-Seq data, we confirmed that the *XIST* expression dosage mirrors the X chromosome number at the single-cell level highlighting a high degree of *XIST* transcriptional homogeneity in case and control samples (Figure 4B and Mendeley data). Next, we correlated the expression of PAR and non-PAR escape genes with that of *XIST*. To this end, we interpolated the levels of expression of the non-PAR escape genes *KDM6A*, *PUDP,* and *EIF2S3* (Figure 4I) and the PAR genes *PPP2R3B* and *SLCA25A6* over cells distributed according to *XIST* levels. Our results indicate that the expression of both PAR and non-PAR escape genes reflects the number of Xs (Figures 4C–H and Supplementary Figure S4C) in an XIST-independent manner (Figure 4I). In fact, high levels of escape genes are detected in cells expressing variable levels of *XIST* (Supplementary Figures S4D,E; Supplementary Table S6). Moreover, to evaluate whether genes that are expressed biallelically upon X chromosome erosion are directly regulated by *XIST*, we profiled their expression at the single-cell level for the mildly eroded 47,XXY KS3#A and the non-eroded 47,XXY KS6#B (Supplementary Figures S4F,G and Mendeley data). Our data indicated that the expression level of both, the reactivated X-linked genes and *XIST* is significantly higher in the eroded line than in the non-eroded line (Supplementary Figures S4F,G). Moreover, none of the reactivated genes biallelically detected in KS3#A significantly correlates with *XIST* expression (Figure 4J; Supplementary Figure S4H). Altogether, these findings imply the existence of an XIST-independent mechanism of reactivation.

Intriguingly, the lncRNA *XACT*, a precocious marker of X chromosome reactivation (Vallot et al., 2015), is barely expressed in KS- and HGA-iPSCs regardless of the X inactivation status. Therefore, the expression of the reactivated genes is not attributable to *XACT* (Supplementary Figure S4I), suggesting that X erosion could occur in an XACT-independent manner in X aneuploid-iPSCs. Additionally, our data demonstrate that high-grade X aneuploidies are not more prone to X erosion than low-grade X aneuploidies. A previous report suggested that fluctuating *XIST* levels and the consequent heterogeneous XCI status could be related to the specific cell cycle state of each individual cell in human fibroblasts (Garieri et al., 2018). By contrast, a study on murine pluripotent stem cells showed that there was no correlation between *XIST* expression and cell cycle phases (Jonkers et al., 2008). Therefore, we applied a previously annotated transcriptomic cell cycle marker gene matrix (Scialdone et al., 2015) to assess the cell cycle stage of each captured iPSC, irrespective of cell line or aneuploidy grade (Supplementary Figures S5A,B). Our data demonstrate that the expression of *XIST*, PAR escape, and non-PAR escape genes is cell cycle-independent in control and KS- and HGA-iPSCs (Supplementary Figures S5C–F).

### The Pre-Existing X Chromosome Inactivation Status of Low- and High-Grade X Aneuploid iPSCs is Retained During Differentiation

**FIGURE 5:**
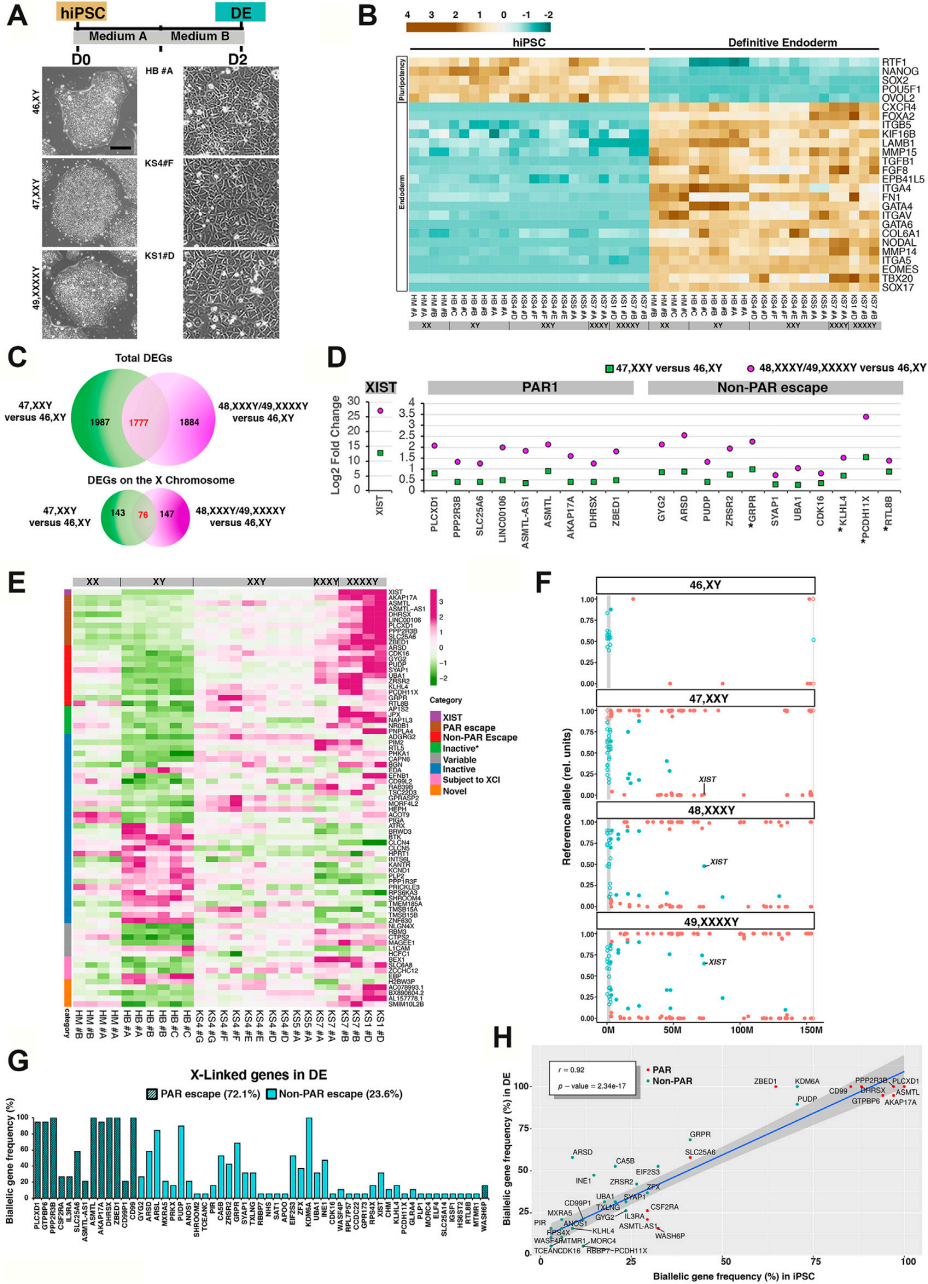
Gene expression profiles and ASE analysis of iPSC-derived definitive endoderm (DE) cells. **(A)** Top: Schematic showing the DE differentiation timeline. Bottom: Representative morphologies of hiPSCs (left) and differentiated DE counterparts (right). Scale bar, 200 µm. **(B)** Heatmap illustrating the FPKM expression of pluripotency genes and DE-specific markers in the indicated hiPSCs and DE cells. **(C)** Venn diagram analysis of global (upper Venn) or X chromosome-restricted (lower Venn) up- and downregulated DEGs from the comparisons 47, KS versus 46,XY and 48/49, HGA versus 46,XY. **(D)** Log_2_ fold change of shared dosage-sensitive X-linked DEGs across the two comparisons of 47,XXY versus 46,XY (green) and 48/49, HGA versus 46,XY (purple). Two groups of DEGs are shown: genes from the PAR1 and non-PAR biallelic genes escaping Xi (Escape) in our iPSC cohort. DEGs indicated by asterisks have been previously described as inactive (*KLHL4*), variable (*GRPR* and *PCDH11X*), or with unknown Xa/Xi status (*RTL8B*) (Tukiainen et al., 2017). **(E)** Heatmap showing the proportional and inversely proportional FPKM expression of X chromosome dosage-sensitive DEGs shared across the two comparisons in PAR1, non-PAR escape, inactive, and novel categories from DE cells across all genotypes. Inactive DEGs marked by the asterisk have been previously described as escape genes. DEGs were defined as variable and subject to XCI accordingly to previous studies (Tukiainen et al., 2017). **(F)** Scatter plot profiles of WES analysis based on allele-specific RNA-Seq of X chromosomes showing the distribution of mono- (orange dots) and biallelic (light blue dots) variant expression status from DE cells in the indicated genotypes. Gray rectangles indicate PAR1 and PAR2. Solid dots show non-PAR genes; open dots indicate PAR genes. The *XIST* allele-specific expression status is displayed. **(G)** Graph showing the biallelic expression frequency [(bars = counts of biallelic detection of the indicated gene/total number of replicates) × 100] for each detected biallelic gene along the X chromosome in DE (*n* = 17 RNA-Seq replicates from nine iPSC clones. Genes located in PARs are indicated with black stripes, and non-PAR genes are identified by light blue color. The means of biallelic gene detection for PARs and non-PAR genes were 72.1% = 23.6%, respectively. Student’s *t-*tests were used to calculate the significance of differences between the number of biallelic genes detected for PAR versus non-PAR genes (*p* = 3.02e-04). **(H)** Scatter plot showing the significant correlation of the identity of biallelic genes in iPSC and DE stages. Red dots label PAR genes; green dots identify non-PAR escape genes. Pearson correlation analysis (*r*) *p-*values are shown.

Previous studies have shown that the X-linked gene inactivation state is retained during the differentiation of human female iPSCs (Patel et al., 2016; Tchieu et al., 2017). However, whether the Xi is retained during the differentiation of iPSCs derived from high-grade X aneuploidies is unknown. Therefore, we differentiated KS and HGA-iPSCs into definitive endoderm (DE), an intermediate progenitor of hepatocytes and beta pancreatic cells. The choice of using DE as an informative differentiated intermediate relies on the consideration that DE cells can be obtained through standardized protocols yielding highly homogenous differentiated cells in a relatively short time window (Pagliuca et al., 2014; Rezania et al., 2014; Astro and Adamo 2018). Additionally, up to 60% of patients with KS develop metabolic syndrome and insulin resistance, both of which are ascribable to inefficient pancreatic and hepatic functions (Bojesen et al., 2006). We successfully differentiated KS- and HGA-iPSCs into DE (Figure 5A and Supplementary Figure S6). Transcriptomic data revealed a highly homogenous differentiation efficiency, confirming the shutdown of pluripotency genes and the concomitant induction of DE markers (Figure 5B and Supplementary Figure S7A). We performed a differential expression analysis as for the iPSCs, comparing 47, KS with 46,XY and 48/49-HGAs with 46,XY. Out of 14,319 expressed genes in DE, we found 3,764 and 3,661 DEGs in 47, and 48/49, HGAs versus the healthy male cohort, respectively. Among them, 1,777 DEGs are commonly dysregulated (Figure 5C and Supplementary Table S7). Restricting the analysis to X-linked genes, we identified 76 common DEGs. As observed in iPSCs, we detected a subset of X-linked dosage-sensitive genes including *XIST*, PAR1 genes (9), and non-PAR escape genes (11) (Figures 5D,E). Gene ontology analysis of the commonly dysregulated X-linked genes revealed a striking enrichment for glycogen and glucan metabolism, immune response and intellectual disabilities, all related to hallmarks of KS (Supplementary Figures S7B,C).

To ascertain whether the pre-existing XCI status detected in the X aneuploid-iPSCs cohort is retained during *in vitro* differentiation, we performed ASE analysis at the DE stage (Figure 5F). Our findings indicate that the frequency of biallelic detection on Xp and Xq in pluripotency is maintained in differentiated cells (Figure 5G) and that the identity of biallelically expressed genes in iPSCs and DE is significantly conserved (Figures 5G,H; Supplementary Figure S7D; Supplementary Table S8). Importantly, KS3#A retained the mild X erosion during differentiation (Supplementary Figures S7E,F).

### Low- and High-Grade X Aneuploidies Affect Global Transcriptional Regulation

**FIGURE 6:**
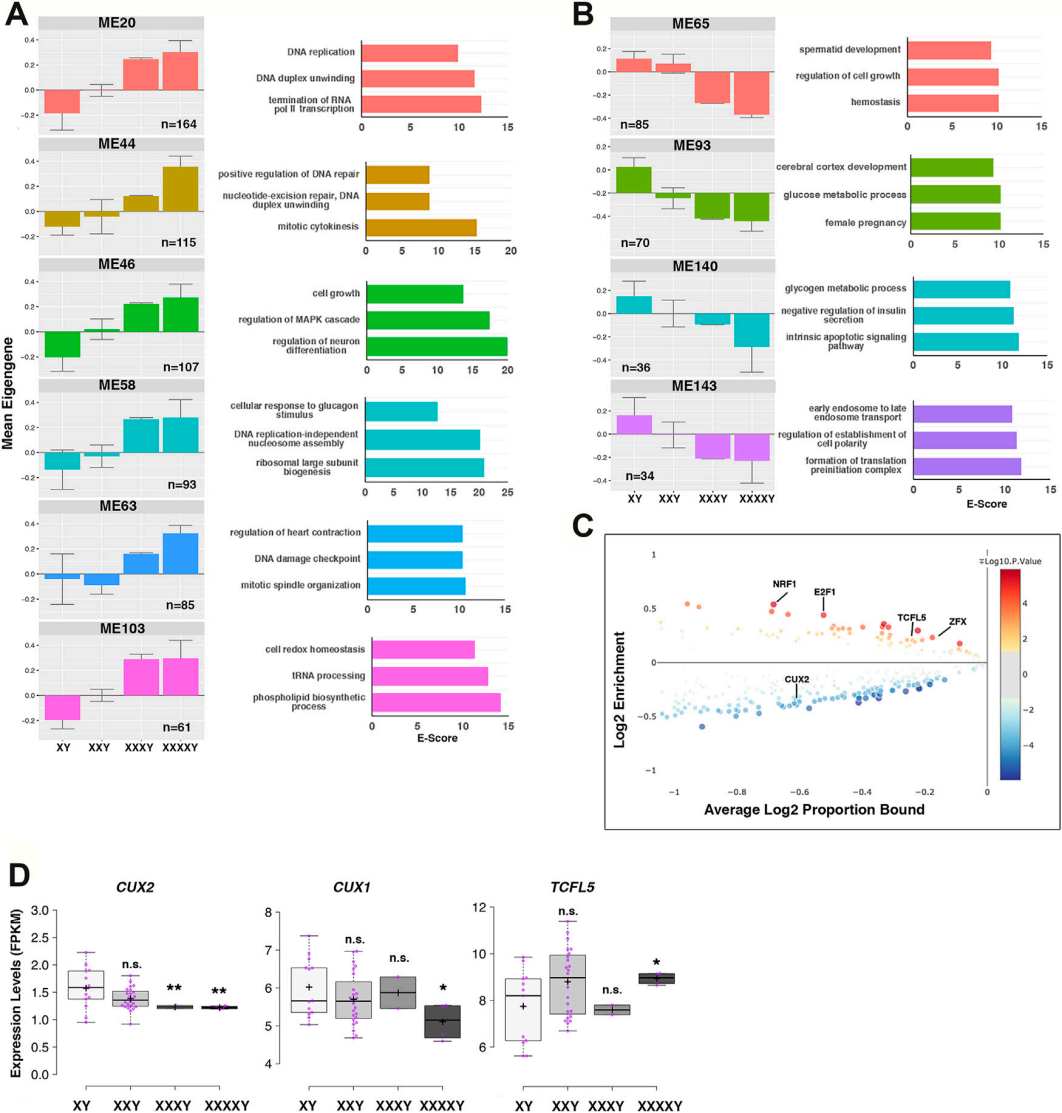
WGCNA of the effects of sex chromosome dosage on the global transcriptome. **(A)** Histogram plots showing the mean expression (±95% confidence) by genotype for six selected proportional X chromosome dosage-dependent gene co-expression modules (left) and enrichment scores (E scores) of the top three Gene Ontology (GO) terms (right) for each of the ten selected supermodules. **(B)** Histogram plots showing the median expression (±95% confidence) by genotype for four inversely proportional X chromosome dosage-dependent gene co-expression modules (left) and enrichment scores (E scores) of the top three GO terms for each module (right). **(C)** CiiiDER enrichment analysis showing the transcription factor binding sites (TFBSs) that are significantly over-represented (greater than zero) and under-represented (less than zero) for the supermodule ME48 compared with the background sequences. The plot shows the proportion of regions bound for each TF. The y-axis shows the enrichment (ratio of proportion bound) and the x-axis shows the average log proportion bound. The sizes and colors of the circles indicate the ±log10(*p* value). **(D)** FPKM expression values of *CUX1*, *CUX2* and *TCFL5* TFs in iPSCs in the indicated karyotypes. Each purple dot represents an RNA-Seq replicate. Centerlines show the medians; box limits indicate the 25t^h^ and 75th percentiles.

**FIGURE 7:**
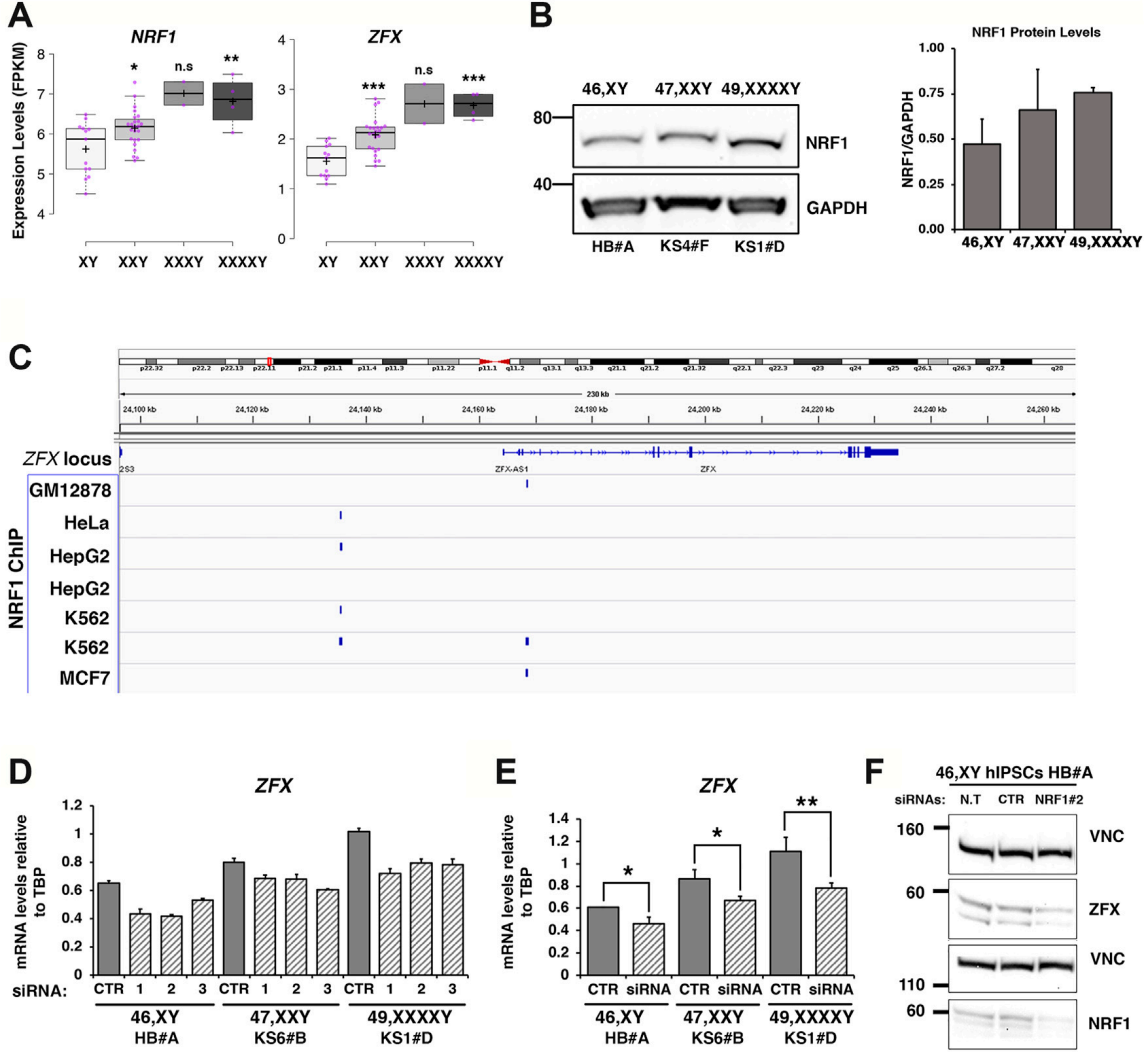
The X dosage-sensitive autosomal TF NRF1 regulates ZFX transcriptional levels. **(A)** FPKM expression values of *NRF1* and *ZFX* TFs in iPSCs in the indicated karyotypes. Each purple dot represents an independent biological RNA-Seq sample. Centerlines show the medians; box limits indicate the 25th and 75th percentile. The significance between the comparison XXY (*n* = 24) versus XY (*n* = 13) and XXXXY (*n* = 4) versus XY (*n* = 13) was calculated using the two tailed Student’s t-test, *p < 0.05; **p < 0.01; ***p < 0.001. **(B)** Lysates from 46, XY (HB#A), 47, XXY (KS4#F), and 49, XXXXY (KS1#D) iPSCs were immunoblotted with the indicated antibodies. Quantification of NRF1 protein levels relative to GAPDH expression was performed using ImageJ (right panel). Error bars are ± standard deviations of two independent experiments. **(C)** NRF1 ChIP-Seq tracks showing the localization of NRF1 at the *ZFX* locus. NRF1 peaks within -30 Kb and +10 Kb from the ZFX gene body are shown (blue rectangles). The ChIP-Seq tracks were downloaded from the ENCODE portal (https://www.encodeproject.org/) with the following identifiers: ENCFF931XAL, ENCFF002CSW, ENCFF003GNY, ENCFF972NIR, ENCFF144PPR, ENCFF493ABN, ENCFF651PWG). **(D)** Graph showing the mRNA expression levels of *ZFX* measured for the indicated iPSC lines and genotypes transfected with a scramble siRNA (CTR) and three different siRNAs targeting *NRF1* (siRNA 1, 2, and 3) (Supplementary Table S13). **(E)** Graph showing the average of two independent knockdown experiments performed on the indicated iPSCs lines transfected with either CTR or NRF1 siRNA number 2. Student’s *t*-tests, *p < 0.05; **p < 0.01; and ***p < 0.001. **(F)** Western Blot showing the down-regulation of NRF1 and ZFX proteins in a 46,XY iPSC line using a siRNA targeting NRF1 (siRNA number 2); Vinculin protein (VNC) was used as loading control. n.t., not transfected, CTR, scramble siRNA.

To ascertain how the X chromosome dosage-sensitive DEGs affect the global transcriptome, we applied a weighted correlation network analysis (WGCNA) (Parikshak et al., 2015) to low- and high-grade X aneuploid and healthy male iPSCs. We identified 147 modules of co-expressed genes. Among them, 38 passed the analysis of variance (ANOVA) statistical test (Supplementary Table S9). We narrowed our analysis to those modules whose expression profiles displayed a linear increasing or decreasing trend mirroring the supernumerary X number, and we selected 10 modules (hereafter “supermodules”) with a significant dosage-dependent pattern (Figures 6A,B). This analysis revealed that X dosage affected disease-relevant processes, including spermatid development (ME65), neuron differentiation, glucose metabolic processes (ME93), and insulin secretion (ME140), all related to the paradigmatic clinical manifestations of KS (Supplementary Table S9). Intriguingly, among the upregulated supermodules, there are several ontology categories associated with DNA duplex unwinding and DNA replication (ME20), regulation of DNA repair and mitotic cytokinesis (ME44), mitotic spindle organization and DNA damage checkpoint (ME63), suggesting a direct correlation with the genomic instability that has led to supernumerary Xs and to the activation of DNA damage response processes (Ma et al., 2012). Next, using an *in silico* approach, we searched the promoters of the genes within the 10 selected supermodules for enriched transcription factor binding motifs (TFBMs). The TFBM analysis identified NRF1, ZFX, CUX1, CUX2, and TCFL5 (Fisher’s exact *p* < 0.05), as overrepresented TFs (identified in at least 7 out of 10 supermodules) whose expression is X chromosome dosage-dependent (Figures 6C,D,7A; Supplementary Figure S8; Supplementary Table S10). The two paralogs *CUX1* and *CUX2* show an expression profile inversely proportional to the X dosage (Figure 6D). Previous knockout and knockdown studies performed in mice and Drosophila have demonstrated that deficiency of these TFs leads to male infertility, intellectual disability, height abnormalities, and scarce body hairs (Ellis et al., 2001; Sinclair et al., 2001; Luong et al., 2002; Platzer et al., 2018), all features strikingly overlapping with the KS phenotype. Moreover, a recurrent *CUX2* missense mutation c.1768G > A; p. (Glu590Lys) has been linked to intellectual disabilities and autism spectrum disorder in humans (Barington et al., 2018; Chatron et al., 2018). Finally, the DNA-binding protein *TCFL5* is involved in spermatogenesis (Maruyama et al., 1998; Siep 2004; Ahn et al., 2017) and contributes to the formation of the sperm flagella in mice (Shi et al., 2013) suggesting a potential role for *TCFL5* in KS infertility.

### NRF1 Is a X-dosage-dependent Autosomal TF Regulating the X-Linked Gene ZFX

Given the striking overrepresentation of NRF1 in eight out of 10 supermodules (Supplementary Figure S8) and its significant upregulation in low- and high-grade X aneuploid iPSCs compared with healthy controls (Figures 7A,B and Supplementary Figure S9), we speculated that NRF1 could be an autosomal regulator of a substantial quota of differentially expressed genes identified in KS- and HGA-iPSCs. Notably, multiple studies have demonstrated an association of NRF1 dysregulation with insulin resistance and type two diabetes (Patti et al., 2003; Cho et al., 2005) as well as autism and schizophrenia (McMeekin et al., 2016), clinical features frequently observed in patients with low- and high-grade X aneuploid patients (Visootsak et al., 2007; Gropman et al., 2010; Cederlöf et al., 2014). We overlapped two published RNA-Seq data obtained from NRF1-overexpressing cells (Liu et al., 2019; Wang et al., 2019) with DEGs identified in both 47,XXY versus 46,XY and 48/49, HGAs versus 46,XY iPSCs (Supplementary Table S11). Approximately 5% (46 out of 862) of KS- and HGA-specific DEGs are shared with NRF1-overexpressing cells. Among the 46 genes, of particular significance is the presence of *ZFX*, a TF already identified as overrepresented in our TFBM analysis and previously indicated as a genome-wide effector of sex chromosome dosage variation (Raznahan et al., 2018; Zhang et al., 2020). Moreover, we analyzed published chromatin immunoprecipitation (ChIP)-Seq experiments for NRF1 in different human cell types (ENCODE portal) and found a significant enrichment of NRF1 binding both within and upstream the *ZFX* gene (Figure 7C). To experimentally prove the functional hierarchy between NRF1 and ZFX we knocked down NRF1 in 49,XXXXY, 47,XXY, and 46, XY iPSC samples and assessed the expression levels of ZFX. Our data demonstrate that ZFX is transcriptionally dependent on NRF1 dosage in all karyotypes (Figures 7D–F). Taken together, these results provide the first experimental evidence of a regulatory role for the X-dosage-dependent autosomal transcription factor NRF1 on the X-linked gene *ZFX*. Our study demonstrates that KS- and HGA-iPSCs can provide disease-relevant insights into the impact of supernumerary X chromosomes on autosomal transcriptional regulation in the pluripotent state.

## Discussion

Our work exploits the recently generated largest cohort of low- and high-grade X aneuploid iPSCs, including genetically matched isogenic, disease, and healthy iPSCs, to shed light on the transcriptional dysregulation associated with supernumerary X chromosomes in early development and disease-relevant cell types. Previous studies on female hiPSCs and hESCs have shown that X erosion is a key limitation for iPSC-based X aneuploidy modeling approaches and suggested that X reactivation could be an intrinsic iPSC characteristic (Vallot et al., 2015) or acquired during *in vitro* culturing (Mekhoubad et al., 2012). We meticulously characterized the status of XCI in iPSCs at low to intermediate passages (p10-p14) and demonstrated that, in the context of tightly regulated XCI, the majority of biallelically expressed genes is located in the PAR1 region, regardless of the number of extra X chromosomes. Our work sets new benchmarks for the characterization of X inactivation in KS- and HGA-derived iPSCs, demonstrating that the canonical dual RNA-FISH for *XIST* and escape genes coupled with H3K27me3 immunostaining is insufficient to score inactive/eroded X chromosomes. In contrast, coupling exome or whole-genome sequencing with RNA-Seq enables reliable discrimination between fully inactive and partially eroded samples (Cantone et al., 2017; Tukiainen et al., 2017; Garieri et al., 2018; Panula et al., 2019; Mandal et al., 2020). We found a subset of PAR1-escape genes, i.e., *PLCXD1*, *GTPBP6*, *PPP2R3B*, *ASMTL*, *AKAP17A*, *DHRSX*, *ZBED1,* and *CD99,* which are biallelically expressed in the majority of low- and high-grade supernumerary X iPSCs, and therefore arguably linked to the progressive severity of KS- and HGAs pathophysiology. Conversely, the transcriptional contribution of PAR2 genes appears to be less prominent, consistent with previous findings obtained in primary PBMCs (Belling et al., 2017; Zhang et al., 2020). However, we can’t exclude that in differentiated derivatives, the contribution of PAR2 genes to the KS and HGA clinical features, could be more relevant. We postulate that PAR1 CNVs could be responsible for shared phenotypic traits between KS and other sex chromosome aneuploidies, such as Jacob syndrome (JS) associated with XYY karyotype (Bishop et al., 2011) and higher grade aneuploidies including 48,XYYY and 49,XYYYY. These phenotypes include infertility, delayed speech and language development and attention deficit disorders as well as a lower than average intelligence quotient (IQ) and an increased incidence of autistic spectrum disorders (Hunter and Quaife 1973; Shanske et al., 1998; Ross et al., 2008; Ross et al., 2010; Bishop et al., 2011; Kim et al., 2013; Demily et al., 2017; Tartaglia et al., 2017). Although the roles of most PAR1 genes have not yet been elucidated, the dysregulation of *SLC25A6* and *CD99* has been linked to frequent KS traits, such as cardiac abnormalities, short QT syndrome (Zitzmann et al., 2015; Skakkebæk et al., 2018), and autoimmune diseases (Lefèvre et al., 2012; Lefèvre et al., 2019).

The evidence that the differentially and biallelically expressed PAR1 genes proportionally mirror the X chromosome dosage in iPSCs and DE, suggests that the impact of PAR1 CNVs is already transcriptionally relevant at the very early stages of X supernumerary embryo development. Moreover, the observation that the XCI status at the iPSC stage is retained upon specification into DE, excludes that drift in the XCI status may occur throughout iPSC *in vitro* differentiation. However, is plausible that a partial reactivation of the inactivated X chromosomes could occur during long-lasting differentiation experiments. Further transcriptomic and epigenomic studies are warranted to address this question.

Previous studies performed using bulk RNA-Seq data have suggested that the reactivation of inactive genes on Xi is not necessarily preceded by *XIST* expression loss or decrease. Here, we show at the single-cell resolution that although *XIST* levels stoichiometrically mirrors X dosage in low- and high-grade X aneuploid iPSCs, the expression of PAR1 and non-PAR escape genes is *XIST*-independent. Moreover, our data demonstrate that high-grade X aneuploidies are not more prone to X erosion than low-grade X aneuploidies.

The differential expression analysis of KS, HGA and control iPSCs highlighted two subsets of DEGs, both X-dosage dependent, but with opposite expression trends. This dual pattern of expression is not restricted to autosomal genes, but is also unexpectedly observed in X-linked genes. A similar counterintuitive finding has been recently reported by analyzing primary human cells with different grades of sexual chromosome aneuploidies versus cells from healthy females (Raznahan et al., 2018; Zhang et al., 2020). Moreover, a trans-acting effect of sex chromosome-sensitive genes on autosomes, which subsequently affects X-linked genes, has also been previously described in maize and Drosophila X aneuploid models (Guo and Birchler 1994; Sun et al., 2013). The WGCNA analysis confirmed the dual expression trend and enabled the identification of supermodules of co-expressed genes displaying an expression profile sensitive to X-chromosome dosage. A TFBM search focusing on TFs over-represented among these supermodules identified NRF1, ZFX, and TCFL5 as TFs whose expression increases proportionally to X dosage, and CUX1 and CUX2 as TFs displaying an inversely proportional expression trend. CUX1 and CUX2 are two exemplary cases of the negative impact of X dosage on autosomal expression. Their downregulation recapitulates a striking number of typical phenotypic traits of low- and high-grade aneuploidies, such as low IQ, infertility, stature abnormalities, muscle hypotonia, and scarce body hair.

NRF1 is a TF gene located on chromosome seven and is physiologically differentially regulated in men and women (Oliva et al., 2020). Our study reveals that NRF1 expression is sex-chromosome dosage dependent in KS- and HGA-iPSCs. Through an integrative transcriptomic and genomic analysis, coupled with a NRF1 knockdown approach, we prove the existence of a functional link between NRF1 and ZFX, an X-linked TF previously indicated as a critical mediator of the impact of X chromosome imbalance on autosomal transcription (Raznahan et al., 2018; Zhang et al., 2020). Importantly, *ZFX* is differentially expressed in our transcriptomic comparison of KS and HGA versus healthy iPSCs, but biallelically expressed only in 20% of the samples. Additionally, *ZFX* is expressed at low levels from Xi, and its transcript abundance is mostly dependent on Xa. Therefore, the upregulation of ZFX in X supernumerary iPSCs is the result of a trans-acting NRF1-mediated regulatory network sensitive to sex chromosome dosage. Thus, we provide the first evidence of an autosomal TF affecting X-linked genes in the context of X polysomy. Collectively, our data demonstrate that the use of iPSCs with full retention of inactive X is crucial for the *in vitro* modeling of X chromosome aneuploidies. Remarkably, our work digs into the transcriptional dysregulation mediated by the X chromosome overdosage on autosomes. A key question remains open: which of the few identified escape genes sensitive to X dosage is the main regulator of the autosomal TF NRF1?

Future studies on isogenic X aneuploid-iPSCs corrected for individual PAR1 and non-PAR escape genes overdosage will allow the dissection of the contribution of each escape gene to the variety of KS and high-grade X aneuploidies clinical features. Further differentiation of KS- and HGA-iPSCs towards disease-relevant lineages will also serve as a powerful cellular platform to study the biological consequences of X polysomy during early development.

## Web Resources

ImageJ, https://imagej.net

qPCR Design and Analysis Application, https://apps.thermofisher.com/apps/spa/#/apps

Social Science Statistics, https://www.socscistatistics.com/

Artificial Intelligence RNA-Seq (AIR) Software, https://transcriptomics.cloud

The R Project, https://www.r-project.org/

FeatureCounts, http://bioinf.wehi.edu.au/featureCounts

DESeq2, https://bioconductor.org/packages/release/bioc/html/DESeq2.html

STAR, https://groups.google.com/d/forum/rna-star

HTSFilter, https://www.bioconductor.org/packages/release/bioc/html/HTSFilter.html

edgeR, https://www.bioconductor.org/packages/release/bioc/html/edgeR.html

EnrichR, http://amp.pharm.mssm.edu/Enrichr/

BBDuk, https://jgi.doe.gov/data-and-tools/bbtools/

SC3, https://www.bioconductor.org/packages/release/bioc/html/SC3.html

Scater, https://www.bioconductor.org/packages/release/bioc/html/scater.html

scImpute, https://github.com/Vivianstats/scImpute

GATK, https://gatk.broadinstitute.org/hc/en-us

WGCNA, https://horvath.genetics.ucla.edu/html/CoexpressionNetwork/Rpackages/WGCNA/

UMI-tools, https://umi-tools.readthedocs.io/en/latest/

## Data Availability

The datasets presented in this study can be found in online repositories. The names of the repository/repositories and accession number(s) can be found below: Gene Expression Omnibus GSE152001. Mendeley data repository: DOI: 10.17632/yc2gpktx9t.1.
