# Supplementary material for: Pseudoautosomal Region 1 Overdosage Affects the Global Transcriptome in iPSCs From Patients With Klinefelter Syndrome and High-Grade X Chromosome Aneuploidies
Source: Front Cell Dev Biol. 2022 Feb 3;9:801597. doi: 10.3389/fcell.2021.801597 (PMC8850648; doi:10.3389/fcell.2021.801597)
Supplement: Supplementary file 2 [file Table1.docx]

**Supplementary Table S1. X-linked STR analysis on case and control fibroblasts of the previously described iPSC cohort** (Fiacco et al. 2021; Alowaysi et al. 2020a; 2020c; 2020b; Fiacco et al. 2020)**.** The STR analysis also allowed to define whether the ancestral non-disjunction event originating the extra X chromosomes in the KS and HGA patients’ cohort (Figure 1A) results from aberrant chromosomal segregation occurring during meiosis I or II. Our data indicate that in four patients (KS2-3-4-5), the X chromosome aneuploidy is consequent to a non-disjunction at meiosis I. Interestingly, the mosaic KS6 results from a non-disjunction event at meiosis II, leading to an XXY karyotype with two identical Xs and a post-zygotic chromosomal loss resulting in a mosaic 46,XY/47,XXY. Notably, the X overdosage in KS1 results from a rare double non-disjunction event at maternal meiosis I and II, as demonstrated by comparing HM and KS1 X-STRs.
