# Supplementary material for: Pseudoautosomal Region 1 Overdosage Affects the Global Transcriptome in iPSCs From Patients With Klinefelter Syndrome and High-Grade X Chromosome Aneuploidies
Source: Front Cell Dev Biol. 2022 Feb 3;9:801597. doi: 10.3389/fcell.2021.801597 (PMC8850648; doi:10.3389/fcell.2021.801597)
Supplement: Supplementary file 5 [file Table2.docx]

**Supplementary Table S2. XIST clouds and KDM6A signals counting from RNA-FISH images and H3K27me3 signals counting from immunofluorescence images.**
