# Supplementary material for: Pseudoautosomal Region 1 Overdosage Affects the Global Transcriptome in iPSCs From Patients With Klinefelter Syndrome and High-Grade X Chromosome Aneuploidies
Source: Front Cell Dev Biol. 2022 Feb 3;9:801597. doi: 10.3389/fcell.2021.801597 (PMC8850648; doi:10.3389/fcell.2021.801597)
Supplement: Supplementary file 6 [file DataSheet1.pdf]

Supplemental Figures and Legends

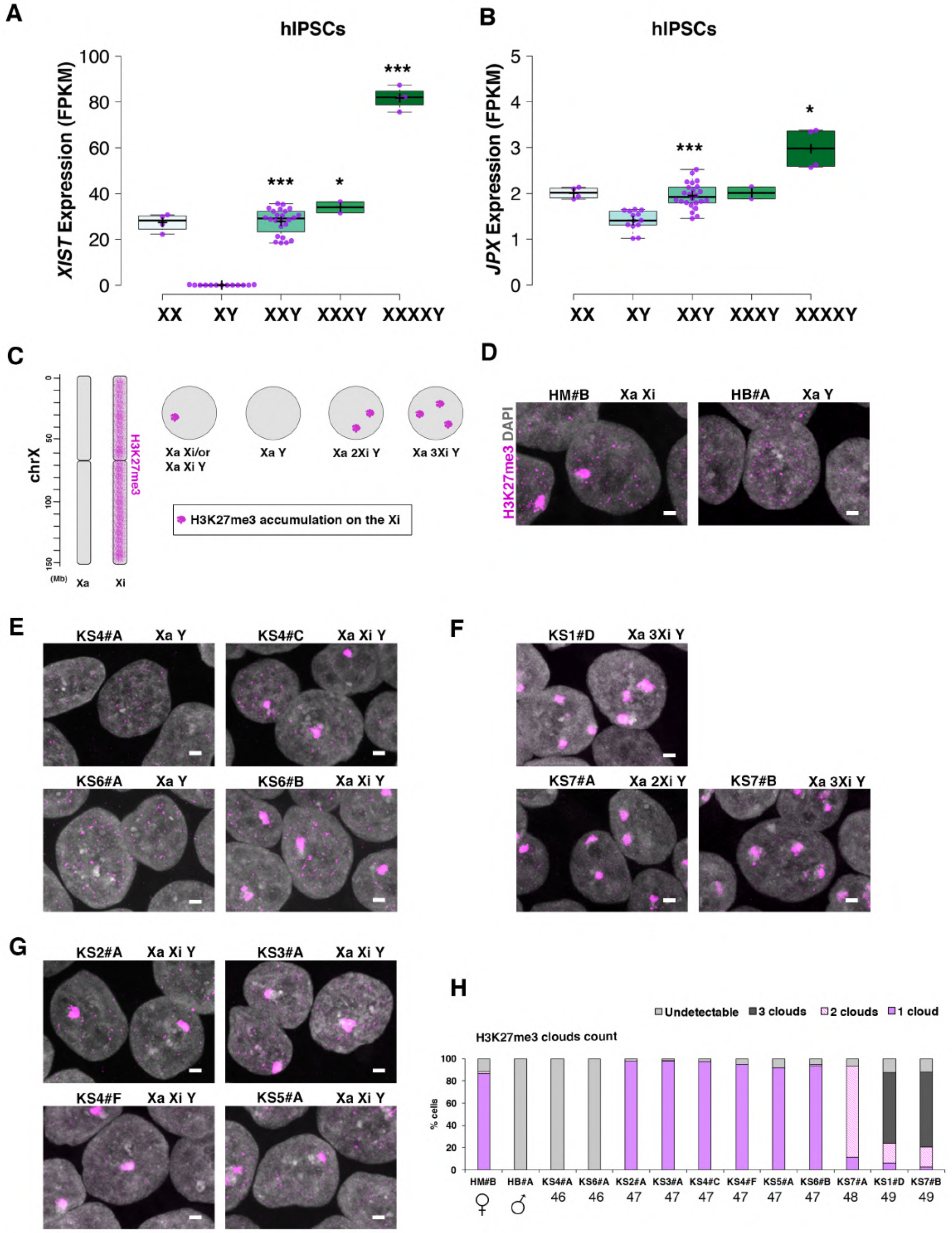

Figure S1

Figure S1: Characterization of X Chromosome inactivation (XCI) in low- and high-grade KS-iPSCs. Box plot for *XIST* (**A**) and *JPX* (**B**) FPKM values at the iPSC stage. Each purple dot represents an RNA-Seq replicate. Centerlines show the median; box limits indicate the 25th and 75th percentile. Whiskers extend 1.5 times the interquartile range from the 25 and 75 percentiles. N= 4, 13, 24, 2, 4 sample points. Two tailed Student's t-*test*, * $p < 0.05$; *** $p < 0.001$. (**C**) Left: Schematic showing the recruitment of the heterochromatic epigenetic mark H3K27me3 on the inactive X. Right: Model for H3K27me3 clouds in euploid and aneuploid KS cells. (**D**-**G**) Representative immunostainings of nuclear H3K27me3 marks (purple) in healthy, low- and high-grade KS-iPSCs were captured with a Zeiss LSM 880 Airyscan confocal laser scanning microscope using a 63X oil objective (Zeiss). DNA was stained with DAPI (grey). Scale bar = 10 µm. (**H**) Percentage of H3K27me3 clouds in approximately 600 nuclei in the indicated iPSC clones.

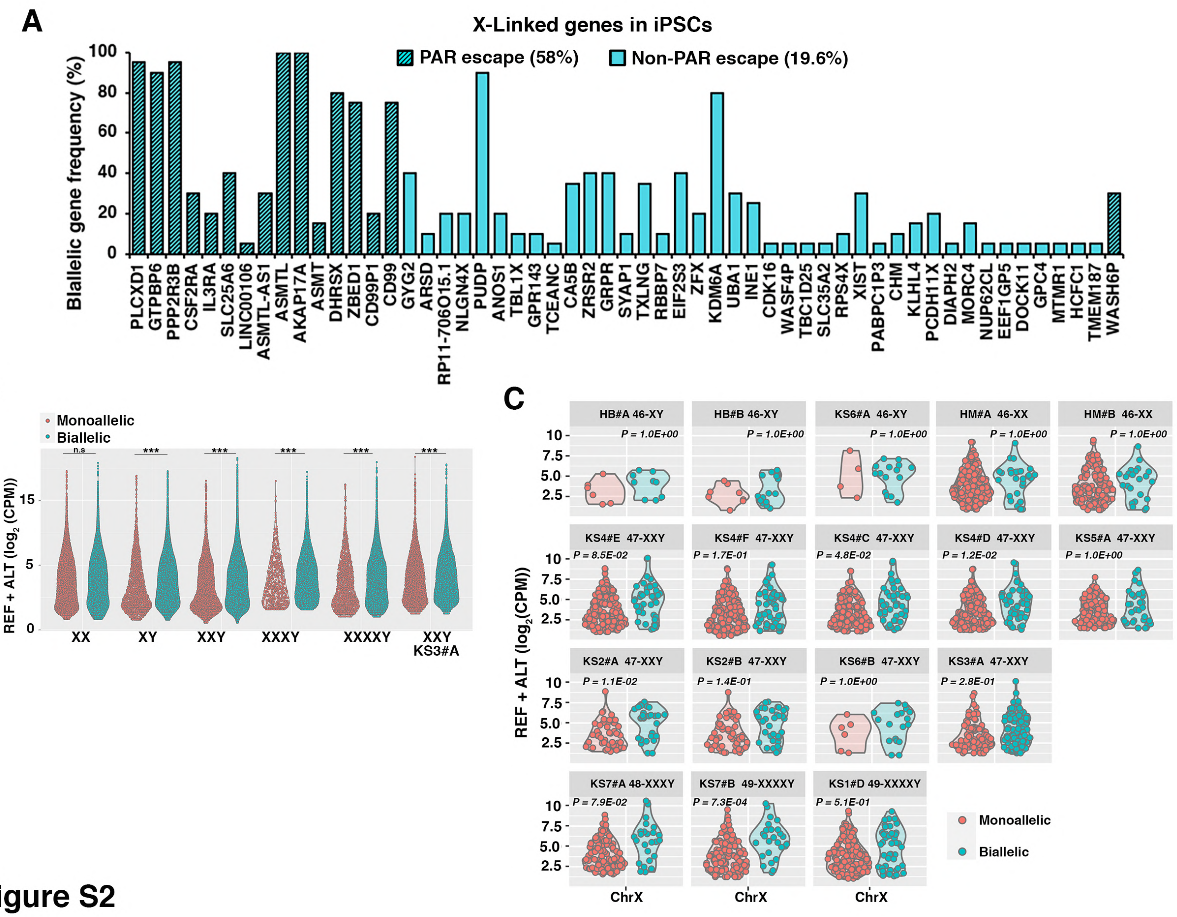

Figure S2

Figure S2: Expression of mono and biallelic genes accordingly to ASE analysis.

A) Graph showing the biallelic expression frequency ([bars = counts of biallelic detection / N. of replicates] *100) for each detected biallelic gene along the X chromosome in iPSCs (n=34 RNA-Seq replicates from 17 iPSC clones (15 males aneuploid iPSCs and 2 female XX-iPSCs in duplicate). Genes located in the PAR regions are indicated with black stripes, non-PAR genes are identified by light-blue color. Mean of biallelic expression detection for PAR = 58% and non-PAR genes = 19.6% is shown. The Student t-test was used to calculate the statistic of the comparison between the number of biallelic detected for PAR versus non-PAR genes (p = 1.00e-03). **B-C**) Violin plots showing the expression in Log_2 CPM units of mono- and biallelic B) autosomal gene variants in all genotypes and C) X-linked genes in all iPSCs. Results from the nonparametric Mann Whitney statistical test are shown. *** $p < 0.001$; n.s = not significant.

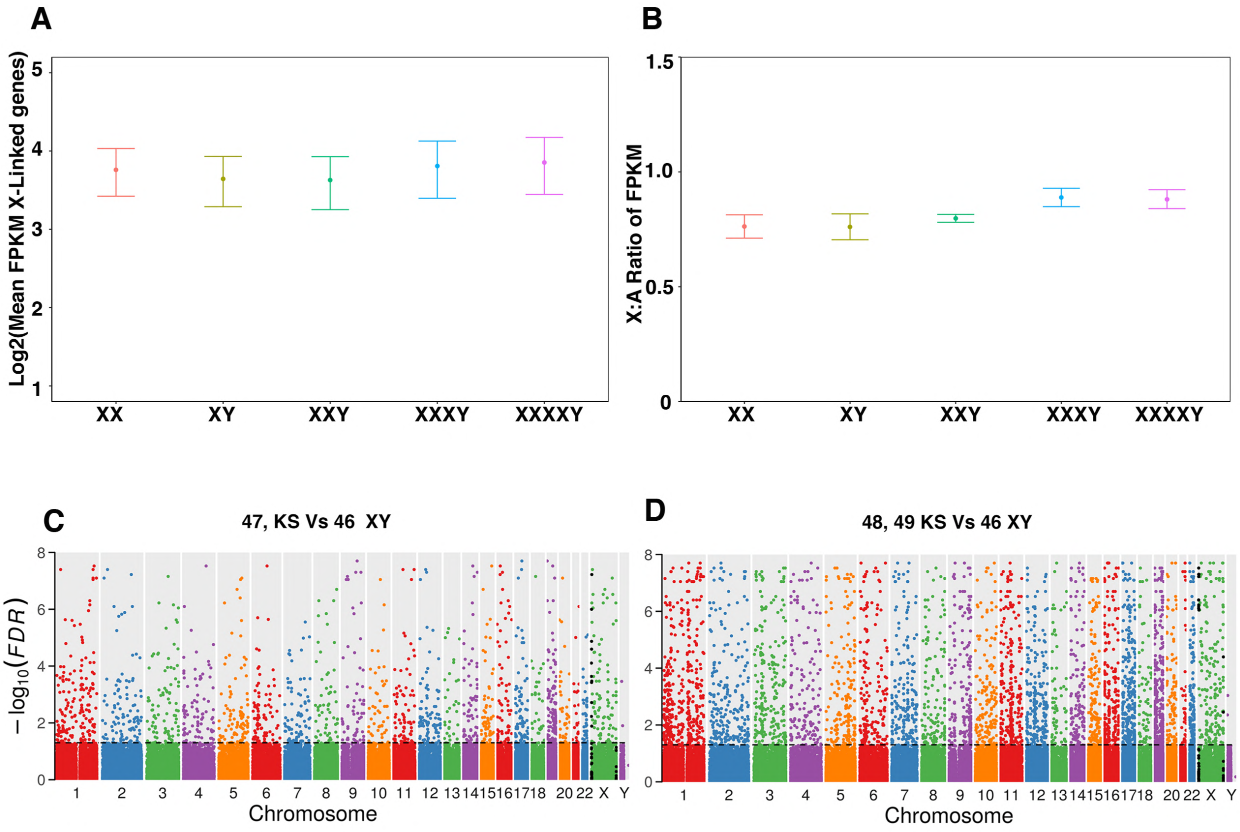

Figure S3

Figure S3: **Transcriptomic profiling of case and control iPSCs.** (**A**) The global expression level of X-linked genes shown as the mean FPKM range in each genotype and (**B**) X:A expression ratio. The ratio of median expression of X-linked and autosomal genes (X:A ratio) was calculated by dividing the mean FPKM values of all X-linked genes (including PAR1 genes) by the mean FPKM expression level of all autosomal genes. Points are the mean of the medians across all the iPSCs according to karyotype and a 95% confidence interval is shown. (**C**,**D**) Manhattan plots showing the -log10 (p-values) of upregulated DEGs in the contrast 47,XXY Vs. male controls 46,XY (C) and 48,XXXY and 49,XXXXY Vs male controls 46,XY iPSCs (D). Genes located in PAR1 and PAR2 regions are represented as black dots. The significance is based on a false discovery rate (FDR) < 0.05 indicated by the horizontal dashed line.

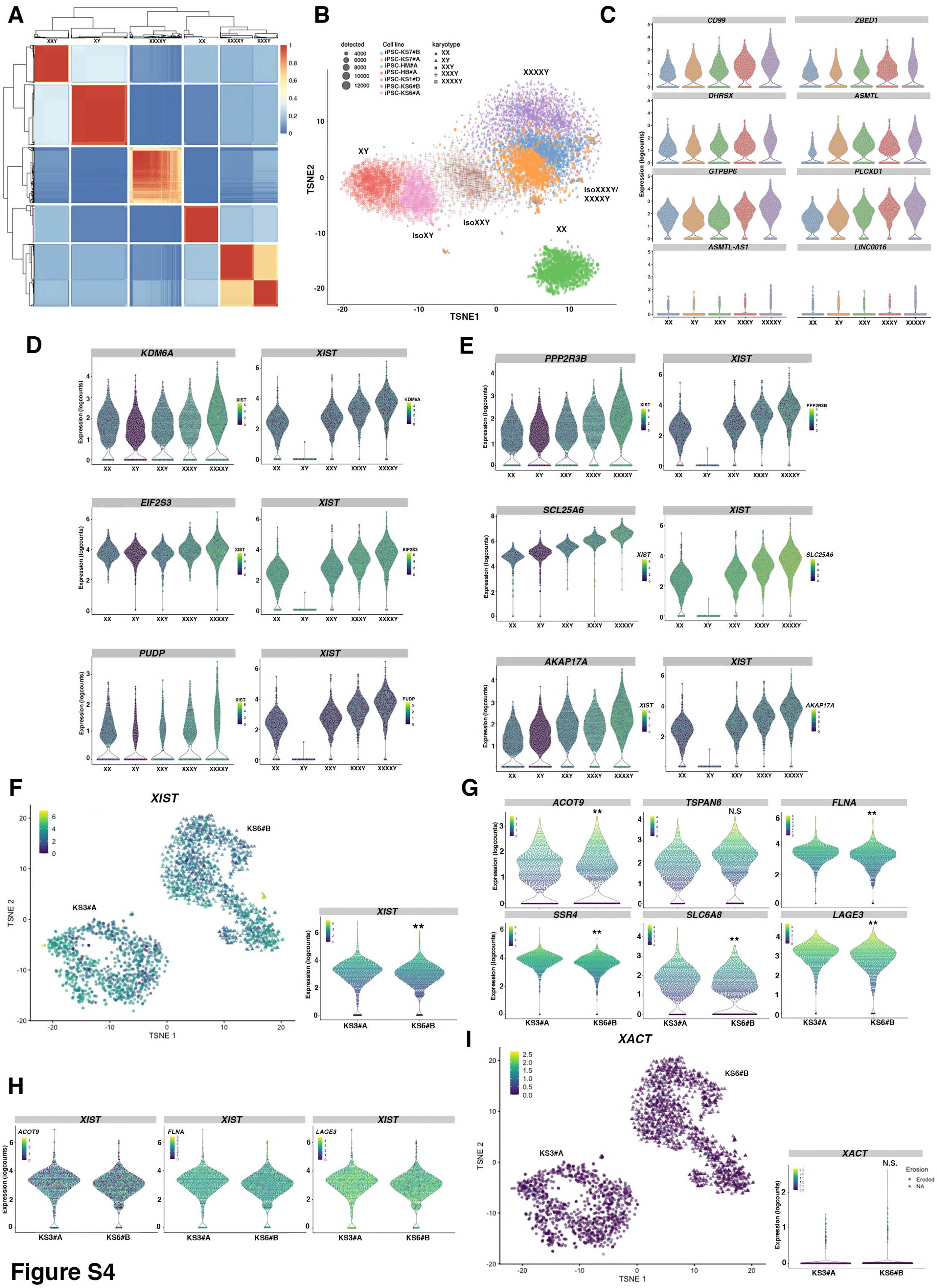
A
XXY
XY
XXXXY
XX
XXXXY
XXXY
B
detected
Cell line
karyotype
iPSC-KS7#B
iPSC-KS7#A
iPSC-HM#A
iPSC-HB#A
iPSC-KS1#D
iPSC-KS6#B
iPSC-KS6#A
XXXXY
XY
IsoXXXY/
XXXXY
IsoXXY
IsoXY
XX
TSNE1
TSNE2
C
CD99
ZBED1
DHRSX
ASMTL
GTPBP6
PLCXD1
ASMTL-AS1
LINC0016
Expression (logcounts)
D
KDM6A
XIST
EIF2S3
PUDP
E
PPP2R3B
SCL25A6
AKAP17A
F
KS6#B
KS3#A
TSNE 1
TSNE 2
G
ACOT9
TSPAN6
FLNA
SSR4
SLC6A8
LAGE3
N.S
H
I
XACT
N.S.
Erosion
Eroded
NA

Figure S4

Figure S4: Single-cell RNA-Seq of KS, controls, and mildly eroded KS3#A iPSCs. (**A**) Hierarchical Clustering of the scRNA-Seq dataset in **Figure 4A** shows the greatest transcriptomic similarity among five groups and each of them identifies one specific genotype. **(B**) t-SNE) plots of the X chromosome-genome transcriptome for seven scRNA-Seq in 46,XX, 46,XY, 47,XXY, 48,XXXY, and 49,XXXXY hiPSCs. Circle sizes represent the number of detected genes/cells. Iso, Isogenenic cell line (**C**) Violin Plots showing the expression profile of genes from the PAR1 region in the indicated genotypes. (**D**,**E)** Left: Violin plots showing cell distribution accordingly to the expression levels of single escape (D) or PAR (E) genes and color-coded accordingly to XIST expression levels. Right: Violin plots showing cell distribution accordingly to the expression levels of XIST and color-coded accordingly to single escape (D) or PAR (E) gene expression levels. (**F**) t-SNE and Violin plots from a mildly eroded (KS#3A) and not eroded (KS6#B) 47,XXY iPSCs showing the expression profile of XIST. (**G**) Expression profiles for the indicated reactivated genes in the 47,XXY eroded and non-eroded iPSCs visualized by Violin Plots. (**H**) Violin plots showing cell distribution accordingly to the expression levels of XIST and color-coded accordingly to single reactivated gene expression. (**I**) t-SNE and Violin plots from a mildly eroded (KS#3A) and not eroded (KS6#B) 47,XXY iPSCs showing the expression profile of XACT. Student's t-*test* ** $p < 0.001$; N.S., not significant.

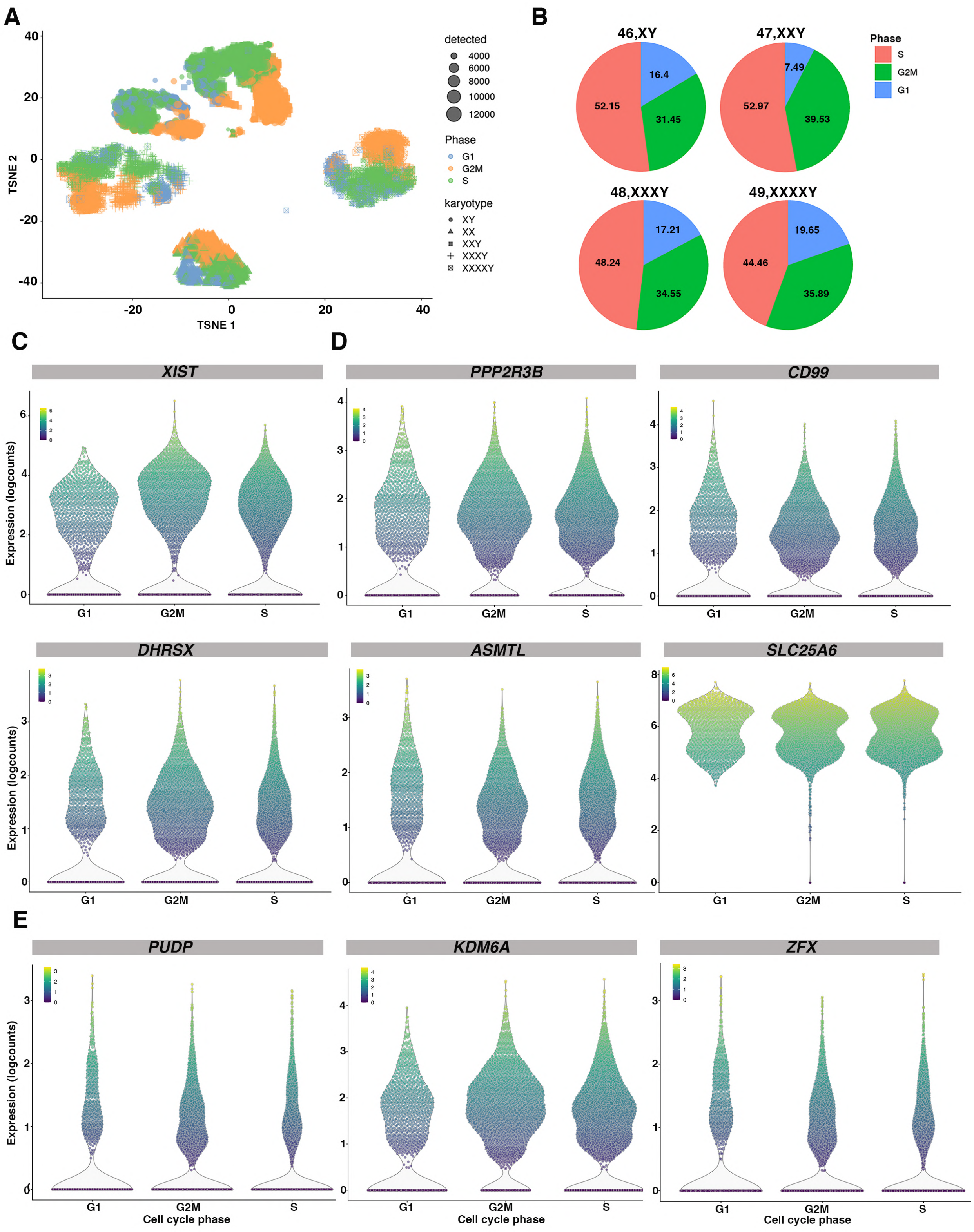
A
TSNE 1
TSNE 2
detected
4000
6000
8000
10000
12000
Phase
G1
G2M
S
karyotype
XY
XX
XXY
XXXY
XXXXY
B
46,XY
47,XXY
48,XXXY
49,XXXXY
52.15
16.4
31.45
52.97
7.49
39.53
48.24
17.21
34.55
44.46
19.65
35.89
C
XIST
D
PPP2R3B
CD99
DHRSX
ASMTL
SLC25A6
E
PUDP
KDM6A
ZFX
Expression (logcounts)
Cell cycle phase

Figure S5

Figure S5: Cell cycle marker analysis on single-cell RNA-Seq of KS and controls iPSCs. (**A**) iPSCs ordered according to the number of expressed genes, karyotype, and cell cycle phase using a t-SNE based analysis. Colors on the t-SNE plot indicate the mitotic cycle phase (G1 blue, G2/M orange, and green for S phase). Marker genes annotated to cell cycle are used from Scialdone et al. (**B**) Pie charts showing the percentage of cells in the indicated cycle phase for 46,XY, 47,XXY, 48,XXXY, and 49,XXXXY karyotypes. (G1 blue, G2/M green, and S phase salmon). (**C-E**) Violin Plots showing the expression profile of (**C**) XIST, (**D**) genes from the PAR1 region, or (**E**) escape genes in cells clustered by cycle phase. Colors in the top bar of the plots show the log expression profile of the indicated gene**.**

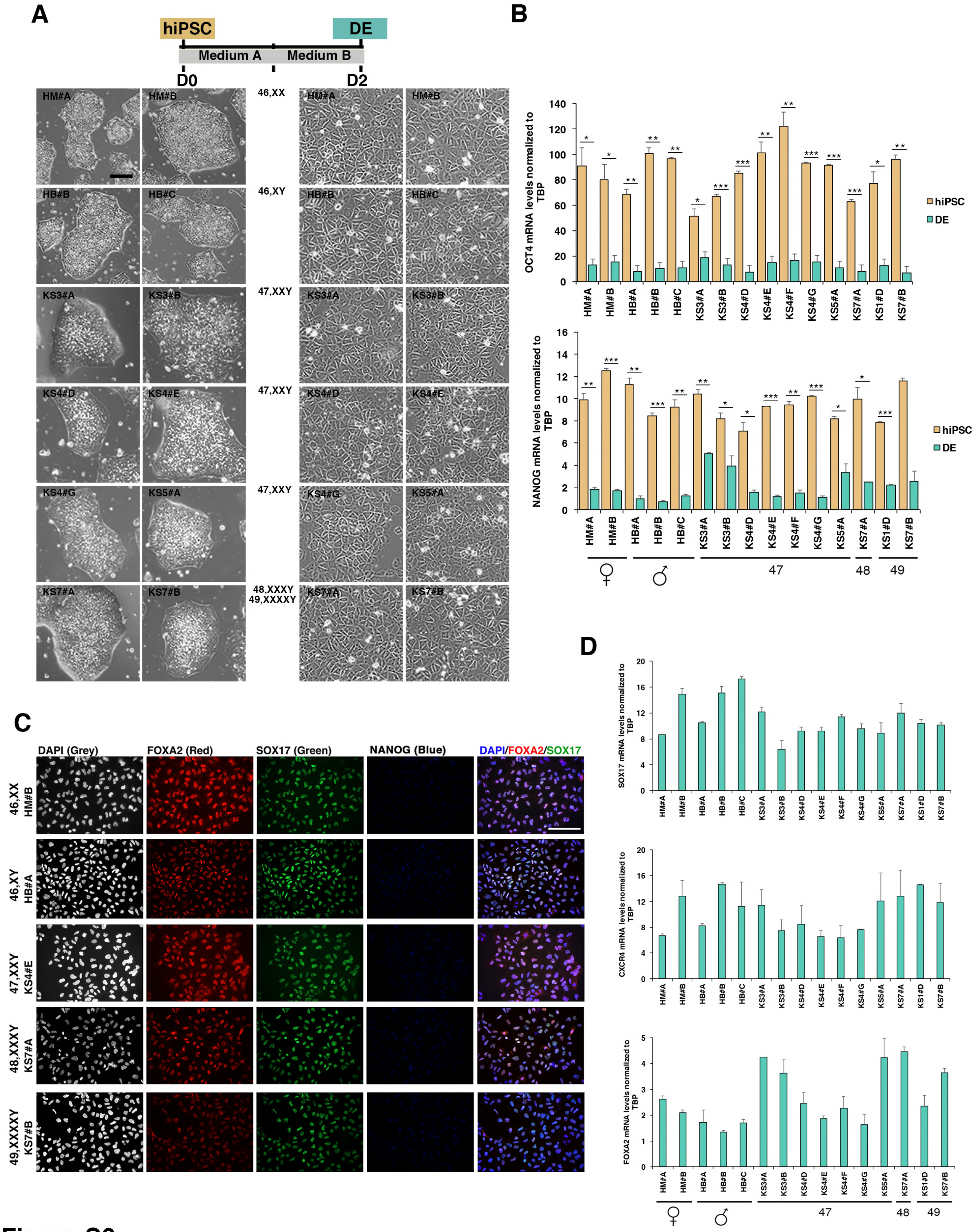
A
hiPSC
DE
Medium A
Medium B
D0
D2
HM#A
HM#B
46,XX
HB#B
HB#C
46,XY
KS3#A
KS3#B
47,XXY
KS4#D
KS4#E
47,XXY
KS4#G
KS5#A
47,XXY
KS7#A
KS7#B
48,XXXY
49,XXXXY
B
OCT4 mRNA levels normalized to TBP
NANOG mRNA levels normalized to TBP
hiPSC
DE
HM#A
HM#B
HB#A
HB#B
HB#C
KS3#A
KS3#B
KS4#D
KS4#E
KS4#F
KS4#G
KS5#A
KS7#A
KS1#D
KS7#B
♀
♂
47
48
49
C
DAPI (Grey)
FOXA2 (Red)
SOX17 (Green)
NANOG (Blue)
DAPI/FOXA2/SOX17
46,XX HM#B
46,XY HB#A
47,XXY KS4#E
48,XXXY KS7#A
49,XXXXY KS7#B
D
SOX17 mRNA levels normalized to TBP
CXCR4 mRNA levels normalized to TBP
FOXA2 mRNA levels normalized to TBP

Figure S6

Figure S6: Validation of KS- and HGA-iPSC differentiation into definitive endoderm (DE) cells. (**A**) Top: Scheme of DE differentiation timeline. Bottom: Representative morphologies of iPSCs (left) and differentiated DE counterparts (right). Scale bar, 200 μm. (**B**, **D**) mRNA expression levels of (B) *OCT4* and *NANOG* and of (D) *SOX17*, *FOXA2*, *CXCR4* measured by Taqman Q-PCR. Data are mean ± SEM (n = 2). Student's t-*test*, **P* < 0.05; ** *P* < 0.01; and *** *P* < 0.001. (**C**) Immunofluorescent staining for DE markers on iPSCs-derived DE cells. Scale bar, 100 μm.

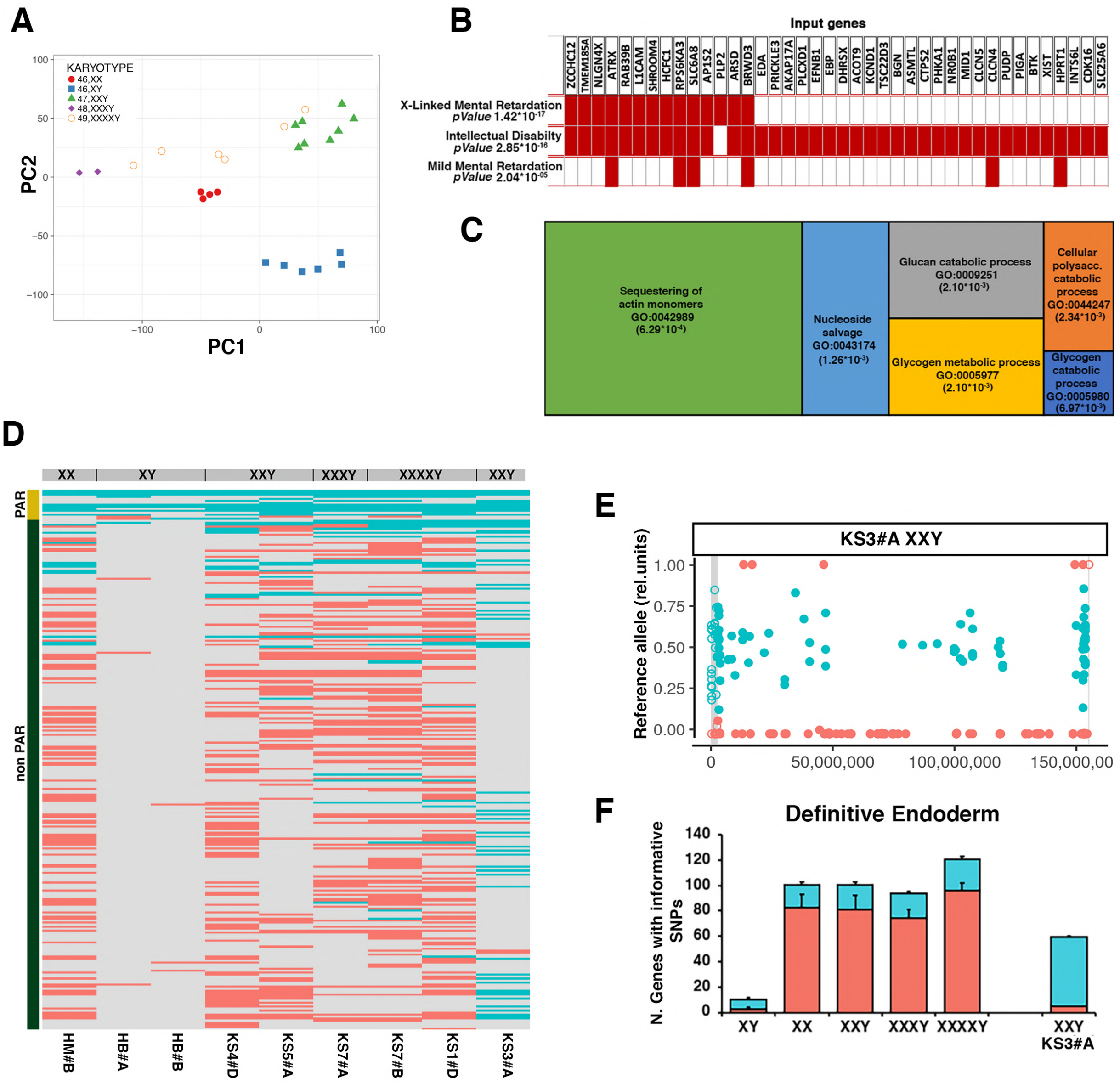

Figure S7

Figure S7: Transcriptomic profiling and ASE analysis on iPSCs-derived DE cells. (**A**) Principal component analysis (PCA) resolves iPSC-derived DE controls from samples with supernumerary X chromosomes. (**B**) DisGeNET and (**C**) EnrichR biological function analysis (lower panel) of shared DEGs in the two comparisons 47, KS Vs 46,XY and 49/48, HGA Vs 46,XY at DE stage. (**D**) Heatmap of mono (orange) and biallelically expressed (light blue) genes in the PAR and non-PAR regions for the indicated iPSCs. The nonparametric Mann-Whitney test shows the statistically significant difference between the active/inactive status of PAR versus non-PAR genes in DE: $p = 2.485e\text{-}15$ for all genotypes, $p < 2.22e\text{-}16$. (**E**) Scatter plot profile of coupled

WES analysis and allele-specific RNA-Seq analysis performed on the X chromosome showing the mono (orange dots) or biallelic (light blue dots) variant expression status in the mildly eroded DE-KS3#A line. Grey rectangles indicate PAR1 (left) and PAR2 (right) regions, respectively. Solid dots show non-PAR genes, open dots indicate PAR genes. **(F)** Histograms showing the number of mono and biallelically expressed genes in DE with informative SNPs. Bars are median ± std of two-three replicates for the indicated genotypes.

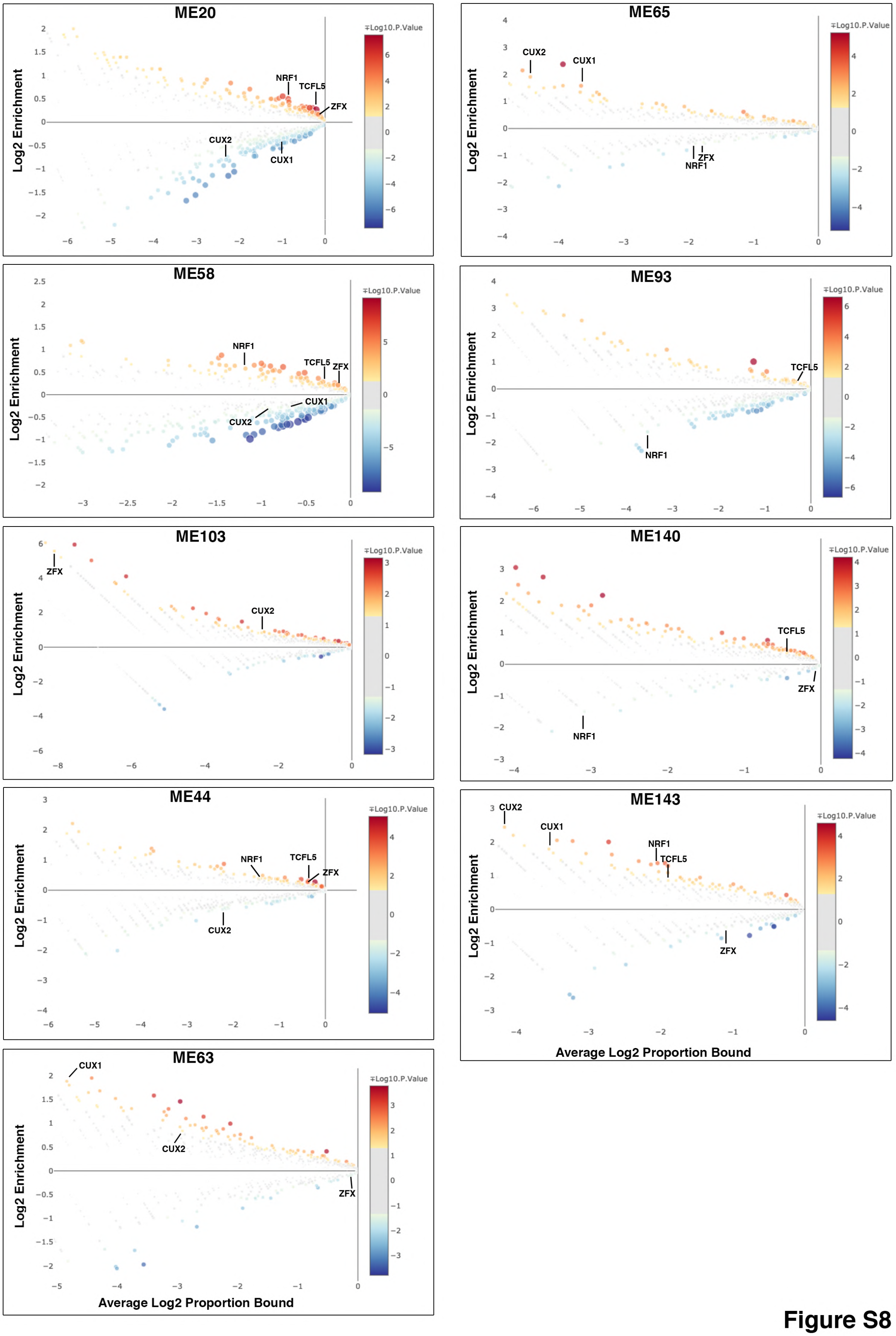
ME20
ME65
ME58
ME93
ME103
ME140
ME44
ME143
ME63
Log2 Enrichment
Average Log2 Proportion Bound
∓Log10.P.Value
NRF1
TCFL5
ZFX
CUX1
CUX2

Figure S8

Figure S8: Transcription Factor Binding site (TFBS) enrichment analysis performed on the 10 supermodules following an X chromosome number dosage-sensitive profile. CiiiDER Enrichment analysis showing the transcription factor binding sites (TFBSs) that are significantly over-represented (greater than zero) and under-represented (less than zero) compared to the background sequences. The plot shows the proportion of regions bound for each TF. The Y-axis shows the enrichment (ratio of proportion bound) and the X-axis the average log proportion bound. The size and color of circles indicate ∓log10(P-value). Are shown the TFs that are more represented in the TFBS analysis: ZFX is recurrent in nine modules, NRF1, CUX2, and TCFL5 in eight modules, and CUX1 is identified in seven out of ten modules.

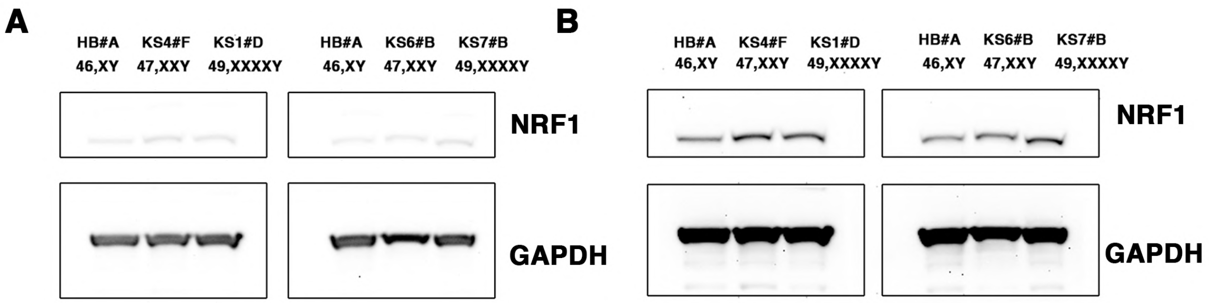

C

	Experiment #1		
	NRF1	GAPDH	NRF1/GAPDH
HB#A	5616.983	9853.518	0.570048484
KS4#F	8724.589	10656.468	0.818713011
KS1#D	7832.468	10600.054	0.738908311

	Experiment #2		
	NRF1	GAPDH	NRF1/GAPDH
HB#A	4750.276	12668.61	0.374964262
KS6#B	6150.811	12286.125	0.500630671
KS7#B	8362.004	10772.296	0.776250857

	Experiment #1	Experiment #2	Average	STDEV
46,XY	0.570048484	0.374964262	0.472506373	0.13794538
47,XXY	0.818713011	0.500630671	0.659671841	0.22491818
49,XXXXY	0.738908311	0.776250857	0.757579584	0.02640517

Figure S9

Figure S9: Western Blot analysis of NRF1 Protein levels. (**A-B**) Immunoblotting of NRF1 and GAPDH (housekeeping gene) from two independent experiments at two different exposures. Right: lysates from 46,XY (HB#A), 47,XXY (KS4#F), and 49,XXXXY (KS1#D). Left: lysates from 46,XY (HB#A), 47,XXY (KS6#B), and 49,XXXXY (KS7#B). (**C**) Quantification of NRF1 protein levels relative to GAPDH expression performed using ImageJ (right panel). The standard deviation (STDEV) of two independent experiments is shown in the table.

Supplementary Table Legends

Supplementary Table S1. X-linked STR analysis on case and control fibroblasts of the previously described iPSC cohort (Fiacco et al. 2021; Alowaysi et al. 2020a; 2020c; 2020b; Fiacco et al. 2020)**.** The STR analysis also allowed to define whether the ancestral non-disjunction event originating the extra X chromosomes in the KS and HGA patients' cohort (Figure 1A) results from aberrant chromosomal segregation occurring during meiosis I or II. Our data indicate that in four patients (KS2-3-4-5), the X chromosome aneuploidy is consequent to a non-disjunction at meiosis I. Interestingly, the mosaic KS6 results from a non-disjunction event at meiosis II, leading to an XXY karyotype with two identical Xs and a post-zygotic chromosomal loss resulting in a mosaic 46,XY/47,XXY. Notably, the X overdosage in KS1 results from a rare double non-disjunction event at maternal meiosis I and II, as demonstrated by comparing HM and KS1 X-STRs.

Supplementary Table S2. XIST clouds and KDM6A signals counting from RNA-FISH images and H3K27me3 signals counting from immunofluorescence images.

Supplementary Table S3. List of biallelic and monoallelic X-Linked genes detected in iPSCs through ASE analysis. The table shows the allelic status of X-linked genes, in which at least one informative SNP was detected. The gene ID, gene name, location, and the category PAR, non-PAR escape accordingly to Tukianen et al. 2017 are shown. Every excel sheet refers to one independent replicate per iPSC line.

Supplementary Table S4. Common DEGs between the comparison 47,XXY Vs. 46,XY and 48,XXXY/49,XXXXY Vs. 46,XY in iPSC. The table includes 862 up and down-regulated DEGs with FDR < 0.05 and LogFC ≥ |0.25| commonly dysregulated in low- and high-grade KS iPSCs, with a dosage-sensitive pattern of expression. The second sheet (X-linked-DEGs) lists 65 common DEGs located on the X chromosome and their biallelic or monoallelic expression status.

Supplementary Table S5. Survey of previously published transcriptomic analysis. The table compares the present study and previous publications (Tukiainen et al. 2017; Oliva et al. 2020; Zhang et al. 2020; Skakkebæk et al. 2018; Raznahan et al. 2018a; Panula et al. 2019; Balaton et al. 2015). The Xa/Xi status of genes in different cell types, the mono- or biallelic gene expression status, the identified X-linked DEGs in healthy males Vs. 47,XXY and sex-biased tissue-specific genes are listed in the table.

Supplementary Table S6. Pearson's correlation statistical test related to Figure 4I. The significance threshold is set at $p < 0.05$. The association between XIST expression and the tested PAR and non-PAR escape genes is not significant independently of the genotype. cor = correlation coefficient.

Supplementary Table S7. RNA-Seq analysis of the differentiated iPSCs into definitive endoderm. List of 1777 up- and downregulated DEGs with FDR < 0.05 and logFC ≥ |0.25| commonly dysregulated in KS- and HGA-hiPSCs, with a dosage-sensitive pattern of expression. The second sheet (X_Linked_DEGs_DE) lists 76 common DEGs located on the X chromosome and their biallelic versus monoallelic expression status.

Supplementary Table S8. List of biallelic and monoallelic X-Linked genes detected in DE through ASE analysis. The table shows the allelic status of X-linked genes, in which at least one informative SNP was detected. The gene ID, gene name, location, and the category PAR, non-PAR escape accordingly to Tukianen et al. 2017 are shown. Every excel sheet refers to one independent replicate per DE sample.

Supplementary Table S9. Gene Co-expression Module WGCNA analysis. Table providing detailed information on the 38 modules defined by WGCNA that passed ANOVA. These include *p-values* for all groups of ME expression module; "top" module genes [expressed by the strength of correlated expression with module eigengene (ME); the complete list of genes for each ME module and supermodule defined by proportional (upregulated) or inverse correlation (downregulated) trends compared to the number of X chromosomes; *p*-values, FDR and enrichment scores (E-score) for the top significant GO term enrichment analysis performed with in-house scripts based on hypergeometric tests (Tian et al.,2017).

Supplementary Table S10. TFBS Enrichment Analysis. List of TFBS enriched in each of the selected ten supermodules. Terms are filtered for their significant score |1.3|. TFBS Enrichment analysis was performed using the Ciider tool.

Supplementary Table S11. RNA-Seq comparative analysis on DEGs induced by NRF1 overexpression. DEGs obtained using RNA-Seq from human cell lines overexpressing NRF1(Liu et al. 2019; Wang et al. 2019) were overlapped with our KS- and HGA-iPSC RNA-Seq datasets.

We have extrapolated a list of 239 commonly dysregulated genes (Sheet 1) in our study and U2OS cells overexpressing NRF1, published in Table S3 by Liu et al., 2019. A total of 116 DEGs were found in common (Sheet 2) between our KS- and HGA-iPSC Cohort and HEK293 cells overexpressing NRF1, obtained from Tables S2 and S3 published by Wang et al.2019. The name of 46 DEGs in common between the three studies are listed in Sheet 3.

Supplementary Table S12. Bulk and single-cell RNA-Sequencing technical Information. Counts of sequenced reads in bulk RNA-Seq uniquely mapped to a single location and number of detected mono- and bi-allelically expressed escape genes. In the second sheet (scRNA-Seq) are reported: the name of the samples, and the number of cells, detected UMIs per cell, and the number of genes per cell for each iPSC line used for the scRNA-Seq. Mean reads per cell and the percentage of reads confidently mapped to the genome are shown.

Supplementary Table S13. List of Taqman gene expression probes, oligos, and siRNAs used in this study.

Supplementary Table S14. List of antibodies and RNA-FISH probes used in this study.
