# Supplementary material for: Pseudoautosomal Region 1 Overdosage Affects the Global Transcriptome in iPSCs From Patients With Klinefelter Syndrome and High-Grade X Chromosome Aneuploidies
Source: Front Cell Dev Biol. 2022 Feb 3;9:801597. doi: 10.3389/fcell.2021.801597 (PMC8850648; doi:10.3389/fcell.2021.801597)
Supplement: Supplementary file 9 [file Table13.docx]

**Table S13a: TaqMan Gene Expression probes**

| **TaqMan Assays** | **Manufacturer** | **Identifier** |
| --- | --- | --- |
| TaqMan qPCR assay: *TBP* | Thermo Fisher Scientific | Cat#Hs00427620_m1 |
| TaqMan qPCR assay: *OCT4* | Thermo Fisher Scientific | Cat#Hs04260367_gH |
| TaqMan qPCR assay: *NANOG* | Thermo Fisher Scientific | Cat#Hs02387400_g1 |
| TaqMan qPCR assay: *XIST* | Thermo Fisher Scientific | Cat#Hs01079824_m1 |
| TaqMan qPCR assay: *SOX17* | Thermo Fisher Scientific | Cat#Hs00751752_s1 |
| TaqMan qPCR assay: *CXCR4* | Thermo Fisher Scientific | Cat#Hs00607978_s1 |
| TaqMan qPCR assay: *FOXA2* | Thermo Fisher Scientific | Cat#Hs00232764_m1 |
| TaqMan qPCR assay: *NRF1* | Thermo Fisher Scientific | Cat#Hs00602161_m1 |

**Table S13b: Oligos used for real-time PCR**

| **Oligo Names** | **Manufacturer** | **Exon location** | **Identifier** |
| --- | --- | --- | --- |
| ZFX | IDT | 6-7 | #228786563 |

**Table S13c: siRNAs used for NRF1 silencing**

| **siRNA Names** | **Manufacturer** | **Sequence** |
| --- | --- | --- |
| siRNA NRF1.1 human | IDT (TriFECTa® RNAi Kit) | 5’-CAUGUGGCUACUUACACCGAGCATA-3’ |
| siRNA NRF1.2 human | IDT (TriFECTa® RNAi Kit) | 5’-AUGCGUUGAGCUACUGACAAACUCA-3’ |
| siRNA NRF1.3 human | IDT (TriFECTa® RNAi Kit) | 5’-AAGCUAUUGUCCUCUGUAUCUCACC-3’ |
| siRNA Control (CTR) human | IDT (TriFECTa® RNAi Kit) | Catalog # 51-01-14-03 |
