# Supplementary material for: Pseudoautosomal Region 1 Overdosage Affects the Global Transcriptome in iPSCs From Patients With Klinefelter Syndrome and High-Grade X Chromosome Aneuploidies
Source: Front Cell Dev Biol. 2022 Feb 3;9:801597. doi: 10.3389/fcell.2021.801597 (PMC8850648; doi:10.3389/fcell.2021.801597)
Supplement: Supplementary file 14 [file Table14.docx]

**Antibodies used for Immunofluorescence and Western Blot.**

| **Antibody** | **Species** | **Dilution** | **Manufacturer** |
| --- | --- | --- | --- |
| Histone H3Me3K27 | Mouse monoclonal | 1:200 | Abcam Cat#ab6002 |
| IgG H&L (Alexa Fluor® 488) | Goat anti mouse | 1:200 | Thermo Fisher Scientific  Cat#A11029 |
| NRF1(D9K6P) | Mouse anti Rabbit | 1:250 | Cell Signalling Technology  Cat#46743S |
| GAPDH (6C5) | Mouse monoclonal | 1:5000 | Abcam  Cat#ab8245 |
| ZFX | Mouse monoclonal | 1:500 | Thermo Fisher Scientific  Cat#PA5-34376 |
| VINCULIN | Mouse Monoclonal | 1:1000 | Thermo Fisher Scientific  Cat#MS-1209P0 |
| FOXA2 | Mouse Monoclonal | 1:100 | Abcam  Cat#ab117542 |
| SOX17 | Mouse Monoclonal | 1:100 | Abcam  Cat#ab84990 |
| NANOG | Rabbit Polyclonal | 1:100 | Abcam  Cat#ab109250 |
| IgG H&L (Alexa Fluor® 488) | Goat anti mouse IgG1 | 1:200 | Thermo Fisher Scientific  Cat# A-21121 |
| IgG H&L (Alexa Fluor® 568) | Goat anti mouse IgG2a | 1:200 | Thermo Fisher Scientific  Cat# A-21134 |
| IgG H&L (Alexa Fluor® 647) | Goat anti rabbit | 1:200 | Thermo Fisher Scientific  Cat# A21244 |

**RNA-FISH probes used in this study.**

| **KDM6A RNA-FISH probes conjugates to Quasar 670**  **Probe Sequence (5' to 3')** |
| --- |
| ggcaggaaatagtttccggaag |
| gaggaaaagagcgatttcgcaa |
| tgtaaaatagcaggcagttgtg |
| taaacaggcacctcgaagatca |
| gggaaaatctgaacacactcac |
| tagaggcaagttaagtccgaaa |
| tagcagtaggcatcacgaacag |
| gacctaaactccgtgaaacttc |
| gcaatcgagatacatgtcaaca |
| aagtcacctaacttcaggtaac |
| ctacggtatttctatgccaaaa |
| aatgacagtgccaacaactttc |
| ataggagcagccattacttaac |
| cgaaggaaaggcttattcacgg |
| cttccaagcttatgaaaggtat |
| ataagcatttaccaggcttatg |
| cctttgttcaatagcttacaga |
| attgcaatcagattttggtcag |
| ggtcccaattacgaagaaagta |
| ctgcaacaataaagctaggtgt |
| agacttatttcaaacttgcgtc |
| gggcttactgaaaagcattatt |
| aattcaagttttctgtatcggt |
| gaagccaattaatacctacgta |
| cactgttcatttttacagcaat |
| ttactgacgcttaaatctttcc |
| gttagaccaacaataatccctc |
| ccaaacttttccacgtgaatta |
| agcattttacacattctcaaca |
| tattcgcagtgaatctatatga |
| aatccgaaatccaatgttaagc |
| gcataactgcagggtttaatat |
| tcatcaaattaccccatatatc |
| tgatgaatcttgtcctaagcag |
| caagaactatatgcggttgcaa |
| tatgttcaggctcacaaagatg |
| aatgtcaggctttacattacac |
| caattcaaacagagggtgcgaa |
| tcgaggactgttaaaagaagga |
| aggaaagccaactcttaagact |
| ttattcaattccattatctgcc |
| ttctgccagaaaggaaaacgtt |
| gttaagggacaagtaagagaca |
| gtttgaataaactgcacacctg |
| tcgggagacgatgagaaatcag |
